# Assessing Global Frailty Scores: Development of a Global Burden of Disease-Frailty Index (GBD-FI)

**DOI:** 10.3390/ijerph17165695

**Published:** 2020-08-06

**Authors:** Mark O’Donovan, Duygu Sezgin, Zubair Kabir, Aaron Liew, Rónán O’Caoimh

**Affiliations:** 1 College of Medicine, Nursing and Health Sciences, National University of Ireland, H91 TK33 Galway, Ireland; mark.odonovan@nuigalway.ie (M.O.); duygu.sezgin@nuigalway.ie (D.S.); aaron.liew@nuigalway.ie (A.L.); 2 HRB Clinical Research Facility Cork, Mercy University Hospital Cork, T12 WE28 Cork City, Ireland; 3 School of Public Health, University College Cork, T12 XF62 Cork City, Ireland; z.kabir@ucc.ie; 4 Department of Endocrinology, Portiuncula University Hospital, Ballinasloe, H53 T971 Country Galway, Ireland; 5 Department of Geriatric Medicine, Mercy University Hospital Cork, T12 WE28 Cork City, Ireland

**Keywords:** frailty, public health, global burden of disease

## Abstract

Frailty is an independent age-associated predictor of morbidity and mortality. Despite this, many countries lack population estimates with large heterogeneity between studies. No population-based standardised metric for frailty is available. We applied the deficit accumulation model of frailty to create a frailty index (FI) using population-level estimates from the Global Burden of Disease (GBD) 2017 study across 195 countries to create a novel GBD frailty index (GBD-FI). Standard FI criteria were applied to all GBD categories to select GBD-FI items. Content validity was assessed by comparing the GBD-FI with a selection of established FIs. Properties including the rate of deficit accumulation with age were examined to assess construct validity. Linear regression models were created to assess if mean GBD-FI scores predicted one-year incident mortality. From all 554 GBD items, 36 were selected for the GBD-FI. Face validity against established FIs was variable. Characteristic properties of a FI—higher mean score for females and a deficit accumulation rate of approximately 0.03 per year, were observed. GBD-FI items were responsible for 19% of total Disability-Adjusted Life Years for those aged ≥70 years in 2017. Country-specific mean GBD-FI scores ranged from 0.14 (China) to 0.19 (Hungary) and were a better predictor of mortality from non-communicable diseases than age, gender, Healthcare Access and Quality Index or Socio-Demographic Index scores. The GBD-FI is a valid measure of frailty at population-level but further external validation is required.

## 1. Introduction

Frailty is a common [1], multi-dimensional [2] condition associated with an increased risk of adverse healthcare outcomes [3]. While there is currently no standard accepted definition of frailty [4], the most widely used approaches are the physical phenotype and the deficit accumulation model. Although measuring different constructs, they are considered equally valid [5]. The accumulation of deficits model conceptualises frailty as a state of vulnerability [6,7] resulting from a breakdown in biological reserve and an inability to maintain homeostasis [8]. This increases the risk of numerous negative health outcomes including reduced quality of life [9] and mortality [10].

Deficit accumulation is estimated statistically through the creation of a frailty index (FI), which includes a variety of health-related items [6,7], selected from available comorbidities, risk factors, functional impairments, psychological and cognitive factors. The FI score for an individual is the sum of the items present or ‘deficits’ as a decimal of the total number of items assessed [6,7]. Guidelines for selecting FI items have been developed with 30–40 items considered the most accurate [11]. Frailty at the population-level is typically quantified taking either the mean FI score or the proportion of individuals above a certain frailty cut-off (e.g., ≥0.25) [11].

The use of a FI to measure frailty is particularly useful for predicting outcomes and stratifying risk at the population-level. However, nationally representative estimates are currently unavailable for many countries and studies are inherently heterogeneous, limiting comparability between countries and regions [12,13]. While FIs have been applied in numerous large datasets [14,15,16,17,18,19] and various clinical settings [20,21,22], to the best of our knowledge, this approach has never been applied to a global population-level dataset such as the Global Burden of Disease (GBD) study.

The GBD represents one of the largest international efforts to globally map morbidity and mortality with the latest iteration (GBD 2017) involving 3,676 collaborators from 146 countries and territories [23]. Comparable population-level estimates of prevalence were computed from thousands of available data sources, which were adjusted using specialised modelling tools including DisMod-MR 2.1 for non-fatal health outcomes [24] and the Ensemble Model or the Cause of Death Ensemble Model for fatal health outcomes [25]. Estimates in the GBD (2017) are available by age and sex for 359 ‘causes’ of disease and injury, 84 ‘risk factors’, 40 ‘impairments’, 17 ‘aetiologies’, and 54 ‘injuries by nature’ in 195 countries and territories.

This study aims to (1) derive a FI for the GBD (GBD-FI), which will inform population-level frailty comparisons, facilitating the monitoring and identification of changes in health performance across comparable settings; (2) examine its face and construct validity by comparing it to existing FIs and (3) test its ability to predict one-year country-level incident mortality from non-communicable diseases based on GBD estimates.

## 2. Materials and Methods

GBD estimates are publicly available through the Global Health Data Exchange providing data by country, age group and biological sex for the years 1990 to 2017 [26]. This study uses the GBD 2017 to develop and internally validate the model performance of the GBD-FI. The GBD 2017 has five categories of heath data: ‘causes’ (*n* = 359), ‘risk factors’ (*n* = 84), ‘impairments’ (*n* = 40), ‘aetiologies’ (*n* = 17) and ‘injuries by nature’ (*n* = 54). A full list of these 554 GBD items and permalinks for downloading the datasets are presented in Table S1. Analyses were carried out using Microsoft Excel Version 2004 (Microsoft, Washington, USA). and SPSS Statistics Version 26 (IBM, New York, NY, USA).

### 2.1. Development of the GBD-FI

The key principles (criteria and considerations) for developing a FI published by Searle et al. [11] were applied as follows:

- *Associated with health status*: Deficits must have the potential to affect health. Hence, attributes that are not linked with health (e.g., greying hair) were not eligible for inclusion [11]. The GBD 2017 only includes items that result in poor health or cause death.
- *Prevalence increases with age:* Deficits should generally increase with age, although reductions at very advanced ages due to survivor effects must be considered [11]. Spearman’s coefficient was calculated for prevalence across the adult age groups (25–29 to ≥95 years). A cut-off of ≥0.7, denoting strong positive correlation with age [27], was applied.
- *Must not saturate too early:* Conditions that increase with age but reach a very high prevalence before old age should be excluded e.g., presbyopia is almost universal by age 55 and thus should be excluded [11]. A cut-off of >80% prevalence has previously been applied to exclude very common conditions in older populations [20]. We also applied this cut-off for the GBD age group of ≥70 years.
- *Cover a range of systems:* If all the index items measure the same characteristic, for example if they all measure cognition, then they are not representative of frailty [11], which is characterised by a decline in function across multiple organ systems [28]. To address this, all sections of the GBD 2017 were considered during item selection including ‘causes’, ‘risk factors’ and ‘impairments’.
- *A single frailty index is to be used serially on the same people:* GBD data represents population-level estimates. However, items that were missing prevalence data were excluded so a consistent list of items was available if serially comparing the same population.
- *Low prevalence:* Items with a low prevalence lack the variability necessary for meaningful comparisons. A cut-off of ≥1% has previously been applied when creating a FI [20]. Since the GBD can be applied to different ages, we took the maximum prevalence from 5-year age groups between 25 and ≥95 years. Causes were excluded if this maximum estimated prevalence was <1%.
- *Redundancy:* The GBD 2017 contains different sections (i.e., ‘causes’, ‘risk factors’ and ‘impairments’) as well as a hierarchical categorisation of items. This means that many of the items are already included within other items. Which ones to include is largely subjective. In general, the more common item was considered more comprehensive and was chosen for inclusion. However, for six items (‘enteric infections’; ‘cardiovascular diseases’; ‘diabetes and kidney diseases’; ‘sense organ diseases’; ‘musculoskeletal disorders’ and ‘unintentional injuries’) the sub-groups were considered more appropriate for inclusion based on clinical judgement and comparison with an existing validated index [17]. The highest order (i.e., level 1) of GBD causes (*n* = 3) were also excluded as these items were considered too broad.

Additionally, the biological plausibility of items was considered by the authors in order to remove items that were inconsistent with existing FIs or were considered unrelated to biological ageing or frailty. Decisions and disagreements were made and settled by consensus amongst the research team. These exclusions included: ‘transport injuries’, ‘other injuries resulting from heat, hot/cold exposure, animals, mechanical forces or a foreign body, ‘other unintentional injury’, an interpersonal violence item, two chemical exposures, four specific dietary risks and high body-mass index. For example, the diet and body mass index items were excluded since they were considered to be more linked with food access and lifestyle than aging-related health. Previous studies have found a U-curve between body mass index and physical frailty where overweight individuals were the least frail [29,30,31]. Details on how GBD items were excluded are presented in the Supplementary Materials (Table S2).

### 2.2. Face Validity of the GBD-FI

The final GBD-FI item list was compared with a selection of 10 validated FIs. Six were FIs selected from a study comparing frailty instruments including the Evaluative Frailty Index for Physical Activity (EFIP) [32]; the Frailty index created by Searle [11]; a frailty index from the Beijing Longitudinal Study of Aging (FIBLSA) [33]; the Comprehensive Geriatric Assessment frailty index (FI-CGA) [21]; a 70-item Frailty Index derived from the Survey of Health, Ageing and Retirement in Europe (SHARE-FI) [34]; and the National Long Term Care Survey Frailty Index (NLTCS) [35]. An additional four indexes where over 70% of the items were biological were selected to illustrate that some indexes, such as the one proposed in this paper, are predominantly composed of comorbidity items. These included the Electronic frailty index (eFI) [17], an 11-item modified Frailty index (mFI) [36], a 32-item multimorbidity frailty index (mFI) [37] and the first laboratory frailty index (FI-LAB) [38]. The items used in each index were categorised into six groups: (1) biological (co-morbidities, signs and symptoms excluding cognitive or mood disorders), (2) cognition, (3) mental wellbeing, (4) nutrition/weight, (5) disability/function, and (6) other (includes self-rated health, hospitalisation, falls/injuries). These groups were based on previously published categories (‘comorbidities’, ‘signs and symptoms’, ‘cognition’, ‘mental well-being’, ‘function’, ‘self-rated health’ and ‘hospitalisation’ [34]). The full list of the items in each FI and their categorisation are provided in Table S3.

### 2.3. Construct Validity and Properties of the GBD-FI

The direction of associations between the FI and other variables were assessed for their consistency with a priori hypotheses for construct validity (i.e., mean index scores are higher for females and increase with age at a rate of approximately 0.02 to 0.03 per year [7,11]). Frailty for a population can be compared using either the mean FI scores, median FI scores or the proportion of the population above a certain frailty cut-off (e.g., ≥0.25) [11]. The GBD represents population-level data, so only the mean FI score could be calculated. Equations relevant to calculating this for a 36-item index are displayed below (Equations (1)–(5)). Taking the formula for calculating an individual FI score (Equation (1)) and the formula for the prevalence of individual deficits (Equation (2)), it follows that a mean FI score for a population is a mean of a mean and can be calculated using several approaches (Equations (3)–(5)). Mean GBD-FI scores for the population were calculated using prevalence estimates according to Equation (5) for the population aged ≥70 in 2017. In addition, scores were calculated using sex-specific and age-specific prevalence estimates to investigate if the GBD-FI possessed the typical FI properties of being higher in women [7] and increasing with age [11]. The rate of deficit accumulation with age was measured by calculating the slope of the line between the natural logarithm of the FI score and age [11]. Location differences in mean GBD-FI scores were also compared by calculating the mean GBD-FI from regional (Europe, Asia, Africa and the Americas *n* = 4) and country-specific (*n* = 195) prevalence estimates available in the GBD 2017. An estimate for those aged 65–94 in the United Kingdom (UK) in 2016 was calculated to compare with published eFI estimates from primary care records [17]. This was calculated by taking the prevalence of each GBD-FI item for 5-years age groups between 65 and 94 and averaging them according to the estimated proportion of the population within each age group using GBD population estimates [39].

(1) $$FI\;score\;for\;one\;person=\frac{Sum\;of\;36\;deficits\;for\;1\;person}{36}$$

(2) $$Prevalence\;estimate\;of\;one\;deficit\;for\;population\;N=\frac{Sum\;of\;1\;deficit\;for\;N\;people}{N}$$

(3) $$Mean\;FI\;for\;population\;N=\frac{Sum\;FI\;scores\;for\;N\;people}{N}$$

(4) $$Combining\;\left(1\right)\;and\;\left(3\right):\;Mean\;FI\;for\;population\;N=\frac{Sum\;36\;deficits\;for\;N\;people}{36\times N}$$

(5) $$Combining\;\left(2\right)\;and\;\left(4\right):\;Mean\;FI\;for\;population\;N=\frac{Sum\;36\;prevalence\;estimates}{36}$$

### 2.4. Measuring YLD, YLL and DALY Estimates for the GBD-FI

The burden of disease is measured in the GBD according to summary measures of population health such as the Years Lived with Disability (YLD) and the Years of Life lost (YLL). The sum of YLD and YLL is Disability-Adjusted Life Years (DALY). YLD is a summary measure of population health used to quantify morbidity, and is calculated by multiplying disease duration by a disability weight (which is a rating of the average severity distribution of each health condition) [40,41]. YLL is a summary measure of population health used to quantify mortality, and is calculated by subtracting the age at death from the highest life expectancy for that age [40]. Taking absolute counts of DALY and YLD from the GBD estimates for those aged ≥70 years in 2017, the absolute number of DALY, YLD and YLL (DALY-YLD) were calculated for the sum of the 36 items. This was compared with the totals from all causes and risk factors (attributable and unattributable global burden) to find what proportion of all YLD, YLL and DALY the GBD-FI items cause.

### 2.5. Outcome Prediction (Internal Validation)

The mean GBD-FI score was calculated by country (*n* = 195) for those aged ≥70 years in 2017 and linear regression models were used to assess whether it could predict mortality (death rate) from non-communicable diseases at the country-level in 2017. Deaths from communicable disease and injuries were not included as these were considered to be heavily confounded by geographic differences unrelated to biological aging (e.g., access to clean drinking water or war). The death rate from non-communicable diseases at country-level was obtained from GBD estimates for 2017. The models were adjusted for four other potential country-level predictors in 2016 including the Healthcare Access and Quality (HAQ) Index, the Socio-Demographic Index (SDI) and population census demographics [39] including the proportion of each population aged ≥85 years and the proportion of each population that were female. The HAQ is a measure of healthcare function for countries and territories calculated from adjusted death rates from 32 causes considered amenable to healthcare intervention based on a previous list by Nolte and McKee [42]. The SDI is a measure of development for countries and territories in the GBD studies based on the Human Development Index, calculated from a composite average of the rankings of the incomes per capita, average educational attainment, and fertility rates of all areas in the GBD study. The adjusted r^2^ value was used to examine whether including the mean GBD-FI score in each model improved the fit of the model, i.e., the predictive ability. The significance of the change in r^2^ was tested by entering the variables in two blocks to obtain a p-value based on the F statistic. The r^2^ value ranges between 0 (explains none of the variance) and 1 (explains all of the variance), and has previously been used to model and predict deaths from non-communicable diseases at the country level [43].

## 3. Results

### 3.1. Development of the GBD-FI

**Figure 1:**
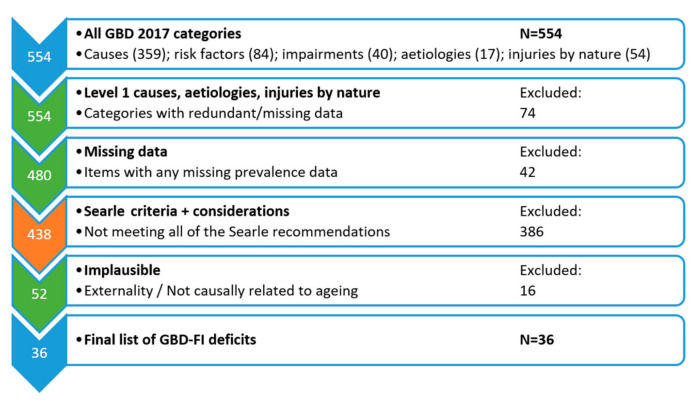
Hierarchical selection of items for the generation of a Global Burden of Disease Frailty Index (GBD-FI), illustrating the number of items excluded according to each criterion applied.

The stepwise selection of items for inclusion within the GBD-FI is presented in Figure 1. The specific reasons for excluding items are presented in detail in Table S2. The initial items excluded were contained in the first hierarchical level of the GBD classification. Categories with redundant or missing data were excluded including ‘causes’ (*n* = 3, too broad), ‘aetiologies’ (*n* = 17, no prevalence data), ‘injuries by nature’ (*n* = 54, accounted for by injury ‘causes’), and other items missing prevalence data (*n* = 42). This left a total of 438 items for further consideration. Applying the principles of Searle et al. (criteria and considerations), a total of 386 items were excluded including 248 that did not correlate with age, 76 with low prevalence, and 62 that were duplicating other items. Finally, 16 items were considered implausible, leaving a total of 36 suitable items for inclusion within the GBD-FI. These 36 GBD-FI items are listed in Table 1 with the proportion (prevalence) of each reported in the GBD (2017) for those aged ≥70 for the global population by sex in 2017. They include 26 non-communicable diseases, 3 metabolic risks factors (high systolic blood pressure, high LDL cholesterol, and low bone mineral density), 3 biological impairments (heart failure, hearing loss, blindness and vision impairment), a single communicable disease (diarrheal diseases from enteric infections), a nutritional deficiency (protein-energy malnutrition), injurious falls, and a function-related risk factor (low physical activity).

### 3.2. Face Validity of the GBD-FI

**Figure 2:**
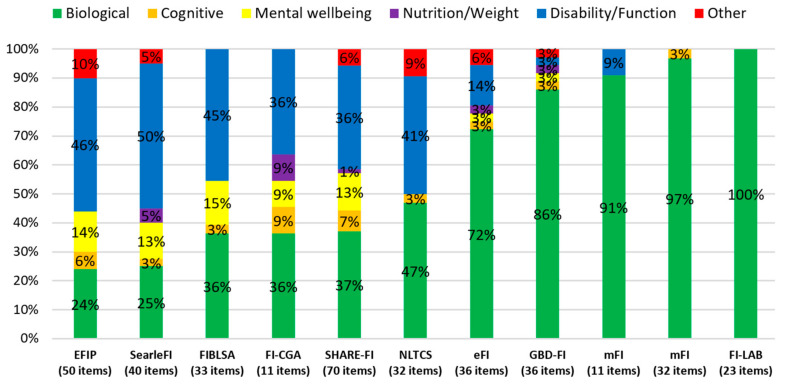
Proportion of deficit types in the Global Burden of Disease Frailty Index (GBD-FI) compared with a range of published Frailty Indexes (FIs). Frailty Index for Physical Activity (EFIP) [32]; Frailty index from Searle et al. [11]; a frailty index from the Beijing Longitudinal Study of Aging (FIBLSA) [33]; the Comprehensive Geriatric Assessment frailty index (FI-CGA) [21]; a 70-item Frailty Index (SHARE-FI) [34] the National Long Term Care Survey Frailty Index (NLTCS) [35]; and the Electronic frailty index (eFI) [17], the 11-item modified Frailty index (mFI) [36], the 32-item multimorbidity frailty index (mFI) [37] and the first laboratory frailty index (FI-LAB) [38]. ‘Other’ refers to self-rated health, hospitalisation, falls and injuries in these FIs.

Figure 2 compares the types of deficits included in the GBD-FI with the items included in a selection of ten published FIs; six with relatively mixed deficits and four with over 70% biological items. The GBD-FI contains 86% biological items (co-morbidities, signs and symptoms) compared with 24% in the EFIP, 72% in the eFI and 100% in the FI-LAB. The GBD-FI illustrates apparent face validity compared with the eFI, which has four disability/functional items compared to one in the GBD-FI. The types of items included in these two indexes were also very similar and covered all major organ systems (Figure S1).

### 3.3. Construct Validity and Properties of the GBD-FI

**Figure 3:**
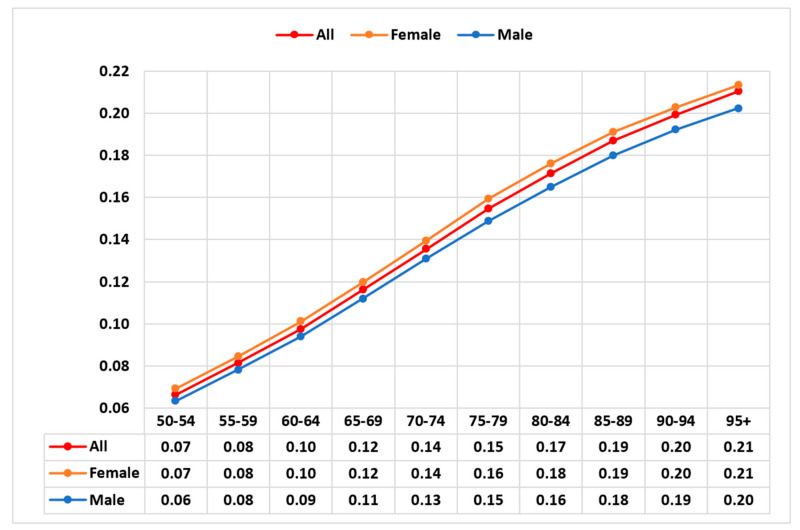
Mean Global Burden of Disease Frailty Index scores by age and sex for the global population of 2017.

The mean GBD-FI score was 0.16 for the global population aged ≥70 in 2017 and was slightly higher in women than men (0.16 vs. 0.15). These sex differences were consistent across all 195 countries/territories. As illustrated in Figure 3, mean GBD-FI scores increased with age. Scores for women ranged from 0.07 in those aged 50–54 years to 0.21 for those aged ≥95 years, increasing from 0.06 to 0.20 for men, respectively. Taking the log of the FI score, the rate of increase in mean GBD-FI scores with age is estimated to be 0.026 per one-year increase from aged 50. This rate increased to 0.028 per year, if the oldest age group (those ≥95 years) was excluded. Examining the rate for those aged between 70–74 and 90–94 inclusive [11], the rate of increase in mean GBD-FI score was lower at 0.02 per year.

**Figure 4:**
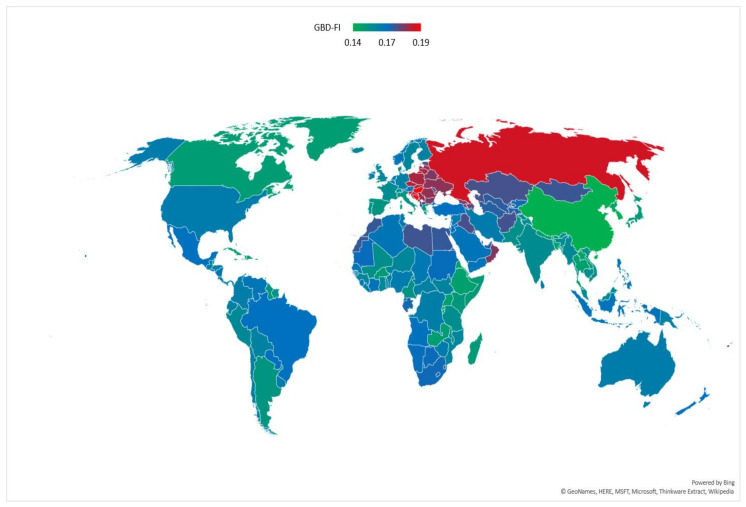
Mean Global Burden of Disease Frailty Index (GBD-FI) scores for 195 countries and territories in the GBD for the global population aged ≥70 years in 2017.

By geographic region, the mean GBD-FI scores ranged from 0.15 in Asia to 0.17 in Europe (Table 2). Considering individual countries and territories (Figure 4), the mean GBD-FI score ranged between 0.13 and 0.19 with the lowest value for China and the highest value for Hungary. Individual deficits varied in prevalence by world region (Table 2) with the largest differences in population proportions seen for blindness, falls and chronic kidney disease. Comparing China and Hungary, 30 of the 36 deficits were higher in Hungary with just over 70% of the difference in the mean GBD-FI score coming from differences in the prevalence of injurious falls (26.8% of difference), high systolic blood pressure (10.3%), low back pain (8.2%), blindness and vision impairment (7.4%), chronic kidney disease (6.4%) diabetes mellitus (5.8%) and chronic respiratory diseases (5.3%). Differences in the prevalence of the 10 cardiovascular-related deficits included in the GBD-FI explained approximately 25% of the difference in mean GBD-FI scores between China and Hungary. Comparing GBD-FI and eFI (from primary care data in the UK) estimates, the mean GBD-FI score for those aged 65–94 in 2016 was 0.142 (0.144 for females and 0.139 for males), similar to the mean eFI score of 0.14 (0.15 for females and 0.13 for males) in the ResearchOne database [39].

### 3.4. Measuring YLD, YLL and DALY Estimates for the GBD-FI

**Figure 5:**
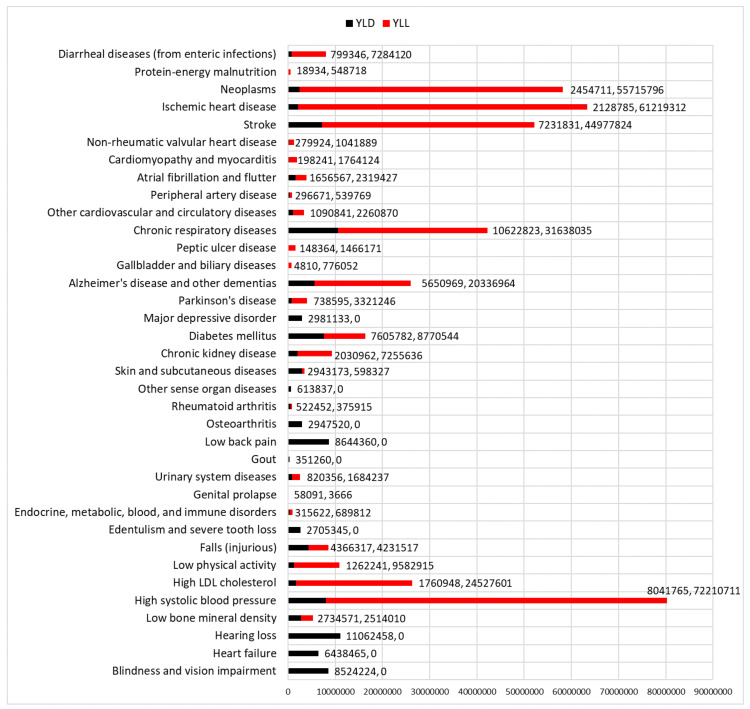
Absolute counts of the Years Lived with Disability (YLD) and the Years of Life Lost (YLL) from the 36 GBD-FI items for the global population aged ≥70 years in 2017. The sum of YLD and YLL is Disability-Adjusted Life years (DALY).

For the global population aged ≥70 in 2017, the 36 GBD-FI items caused 471,269,037 DALY, 110,052,294 YLD and 361,216,743 YLL. These estimates were approximately 19% of total DALY from all causes and risk factors available in the GBD, 13% of the total YLD and 22% of the total YLL. The YLD and YLL estimates of each of the 36 items are presented in Figure 5.

### 3.5. Outcome Prediction (Internal Validation)

We then examined if the country-level mean GBD-FI score for 2016 was a useful predicter of incident mortality from non-communicable diseases at the country-level (*n* = 195) in 2017, compared with four other country-level predictors. The number of deaths from non-communicable diseases per 100,000 ranged between 2851 in Kuwait and 10,390 in Uzbekistan. As illustrated in Table 3, the mean GBD-FI score explained more of the variation in death rates from non-communicable diseases between countries (adjusted r^2^ = 0.143) than either the proportion of females (adjusted r^2^ = 0.068), a measure of development (SDI index adjusted r^2^ = 0.056), a measure of health care function (HAQ index adjusted r^2^ = 0.111) or the proportion aged ≥85 years (adjusted r^2^ = 0.141). For a model containing all four variables (age, sex, SDI, and HAQ), the explanatory power was increased by adding mean GBD-FI score with the adjusted r^2^ increasing from 0.270 to 0.396 (*p* < 0.001). The GBD-FI score was positively associated with the number of deaths from non-communicable diseases such that each 0.01 unit increase in mean GBD-FI score was associated with a 407 (95% CI: 269–546) per 100,000 increase in the number of non-communicable deaths. This remained similar after adjusting for all four confounders with a 405 (95% CI: 279–530) per 100,000 increase.

## 4. Discussion

Age-related diseases were estimated to account for over half (51.3%, 95% uncertainty interval 48.5–53.9%) of all burden of disease (DALY) in 2017, placing a significant impact on limited healthcare resources [44]. In this analysis, we used an accumulation of deficits approach to generate a 36-item FI from age-related diseases, causes of injury, risk factors and impairments in the GBD 2017, intended to act as a global surrogate measure for frailty at the population-level. These items include infectious diarrheal diseases, protein-energy malnutrition, twenty-six non-communicable diseases, injurious falls, low physical activity, three metabolic risks and three biological impairments.

The mean GBD-FI score was 0.16 globally for adults aged ≥70 in 2017, similar to the range of mean scores (between 0.14 and 0.16) observed in other studies applying FIs in Hong Kong [45], the UK [17] and Canada [46]. For example, the mean score was very similar to the mean eFI score in the UK, produced from a nationally representative sample in primary care aged 65–95 [17]. The mean GBD-FI score for those aged 65–94 in the UK in 2016 was 0.14 (0.14 women, 0.14 men). The mean eFI score, derived from two separate datasets was similar but with larger sex differences; the ResearchOne primary care database had a mean score of 0.14 (0.13 men, 0.15 women) compared with 0.15 (0.14 men, 0.16 women) for the THIN database [17]. The mean GBD-FI score was higher in Europe (0.17) than in Africa (0.16), America (0.16) or Asia (0.15). Mean scores ranged from 0.14 in China to 0.19 in Hungary. These differences were due to variations in the prevalence of numerous deficits, although cardiovascular disease or injurious falls were major contributors. Prevalence of and mortality from cardiovascular diseases vary globally but are generally higher in Eastern Europe and Central Asia [47,48]. Rates of falling are also noted to be higher in Eastern European countries and Russia compared to other countries in the GBD [49] with differences in wealth, expenditure on older persons care and intrinsic falls risks considered important contributory factors [50].

The GBD-FI shows typical properties of a FI that suggest it has content validity. For example, it has higher mean scores in females, as well as mean scores that increase with age [7,11]. Mean GBD-FI scores were found to increase with a distinctive rate of deficit accumulation of approximately 0.03 per year [7,11]. This is within the expected range seen with other FIs, which report mean scores between 0.02 to 0.03 [11,16]. Examining the rate of increase per year for those aged 70–74 and 90–94, the rate was 0.02/year. This is similar to the index developed by Searle et al. [11], which showed an increase of 0.02 per year for those aged 70 to 92. However, other properties of FIs such as the tendency to have a right-skewed distribution of scores and a maximum 99 percentile score of less than 0.7 could not be demonstrated in this population-level study and need confirmation in individual-level datasets. Further research is also required to establish whether the GBD-FI is a good predictor of adverse health outcomes in individuals. According to GBD estimates, the 36 items included in the GBD-FI cause about 13% of all YLD (time spent in poor health) and 22% of YLL (prematurity of death) in those aged ≥70 years, which suggests it would be a good predictor. This study also examined the internal predictive validity of the GBD-FI; the addition of mean GBD-FI scores to models containing country-level measures of demographics, development and healthcare improved the ability of the model to predict mortality from non-communicable diseases. The GBD-FI could predict 14.3% of the variance in non-communicable disease mortality globally, which was higher than the SDI, HAQ index or differences in age/sex proportions. It is also higher than a previous model created using socioeconomic variables alone (r^2^ = 11.3%) [43].

Despite behaving like a FI, the authors acknowledge that the GBD-FI items predominantly consist of comorbidities. While future iterations of the GBD study may be able to facilitate the inclusion of measures of function and activity (e.g., mobility), their absence is a current limitation of the global data. The GBD-FI is, however, not unique in terms of this biological-focus and further research is needed to measure the agreement between FIs composed of different numbers of comorbidity and disability items, i.e., those with different proportions of deficits highlighted in Figure 2. Although frailty, multi-morbidly and disability are distinct [51,52], disease and function reasonably correlate [53,54] and have a similar ability to predict the frailty phenotype [55]. For example, many GBD-FI items (diseases) overlap with disability and function, including poor eyesight, hearing loss and lower back pain are related to functional loss and co-morbidities such as Alzheimer’s and Parkinson’s disease impair function and signal disability. This suggests that the GBD-FI deficits, while predominantly co-morbidities, signs and symptoms, may still be a good indicator of overall deficit accumulation. A further major limitation of the GBD data is that it is composed of population-level estimates meaning that only mean GBD-FI scores for the population could be calculated and not the proportion of individuals scoring a GBD-FI score above a certain cut-off (e.g., 0.25, i.e., having ≥9/36 items). Mean scores are sensitive to outliers (i.e., individual deficits with large prevalence differences) and as a result higher scores may not necessarily represent higher levels of frailty (≥9/36 deficits i.e. ≥0.25 in an individual), which depends on the distribution of said deficits. This concern is highlighted by comparing China with Hungary, where there was a marked difference in the prevalence of a single item, falls (10% vs. 81%, respectively). However, on further examination, it was observed that this caused only 27% of the total mean difference in GBD-FI scores and that 30 of the 36 deficits were more prevalent in Hungary. Studies across several countries have found that the distribution of FI scores for individual community-dwellers to be very similar and best fitted by either a gamma or Weibull distribution [11,45]. Given the distribution of GBD-FI scores for individuals is the same globally, then higher mean GBD-FI scores would be expected to show higher frailty prevalence (≥9/36 deficits in an individual).

In addition to the above limitations, there are several other potential limitations in relation to the subjectivity and philosophy of item selection in the development of any FI. Some items were excluded without a formal test of agreement and were based on clinical judgment. Others such as “diarrheal diseases” were included as they met the inclusion criteria, despite being an acute infectious disease. While not a chronic disease, these can exacerbate the impact of ageing on a frail population [56]. Further, these remain an important and relatively common cause of morbidity and mortality among older adults at a global level, particularly *C. difficile*, which is often chronic in nature [57]. In addition, a number of items were excluded where they were estimated to be below 1% prevalence for all adult age groups globally. While this prevalence cut-off is commonly applied when creating a FI, the GBD data consists of estimates and not objective measures, and prevalence varies by country/region. While the principles of Searle et al. [11] are broadly considered a standardised approach to construct a FI, no ‘gold standard’ method exists and the approach is somewhat open to interpretation. We also included biological plausibility as a criterion, which has been used by other researchers to help rationalise and limit the number of deficits included [58]. While each item was discussed by the research team and evidence sought to support each decision, there is still the potential for selection and information bias. By including predominantly age-associated co-morbidities as items, the GBD-FI may be regarded by proponents of the physical phenotype as more of an assessment of chronic disease states rather than frailty or, indeed, ageing [59]. Nevertheless, the primary goal of such an index in the context of global ageing and disease burden is to characterise frailty as a risk state for adverse outcomes and facilitate comparisons, something we suggest the GBD-FI, as with all FIs, can demonstrate [10,13]. The addition of mean GBD-FI scores to regression modelling improved the fit for predicting mortality, explaining more of the differences in death rates between countries. Although only improving the model modestly, this was at country-level and was greater than the effect of the other included predictors (age, sex, SDI, or HAQ index). This suggests that the GBD-FI should be able to predict negative outcomes between individuals, and may be more important than sociodemographic variables.

## 5. Conclusions

In summary, we developed a 36-item GBD-FI using age-related diseases, causes of injury, risk factors and impairments from within the GBD framework using a novel population-level approach. Since the GBD-FI is similar to existing indexes such as the eFI and displays many typical FI properties, it may represent the first globally comparable estimates of mean frailty scores for countries and territories in the GBD. Given the comprehensiveness of GBD data [24] and the absence of frailty estimates for many countries [12,13], this approach may be useful for monitoring and comparing frailty globally. Given the increased recognition and awareness of frailty as a significant public health challenge [3,60], policy-makers and healthcare planners will require such comparative estimates to support effective policy and resource allocation, especially where formal assessment of frailty is unfeasible. Further research is required to validate the GBD-FI against existing FIs and other frailty assessment tools.

## Figures and Tables

**Table 1:** Global prevalence (%) estimates of individual deficits (*n* = 36) included in the GBD-FI for those aged ≥70 in 2017, globally and by sex.

GBD-FI Items (*n* = 36)	GBD Grouping	GBD Level ^5^	All	Female	Male
Diarrheal diseases (from enteric infections)	Communicable	3	1.8%	1.9%	1.8%
Protein-energy malnutrition	Nutritional	3	1.2%	1.1%	1.3%
Neoplasms	Non-communicable	2	6.3%	5.1%	7.8%
Ischemic heart disease	Non-communicable	3	12.3%	10.9%	14.0%
Stroke	Non-communicable	3	8.4%	8.3%	8.5%
Non-rheumatic valvular heart disease	Non-communicable	3	3.6%	3.6%	3.5%
Cardiomyopathy and myocarditis	Non-communicable	3	0.6%	0.6%	0.6%
Atrial fibrillation and flutter	Non-communicable	3	5.1%	4.6%	5.7%
Peripheral artery disease	Non-communicable	3	12.0%	12.2%	11.7%
Other cardiovascular and circulatory diseases ^1^	Non-communicable	3	5.0%	4.3%	5.9%
Chronic respiratory diseases	Non-communicable	3	26.1%	25.7%	26.5%
Peptic ulcer disease	Non-communicable	3	0.7%	0.8%	0.7%
Gallbladder and biliary diseases	Non-communicable	3	1.1%	1.2%	1.0%
Alzheimer’s disease and other dementias	Non-communicable	3	8.6%	9.7%	7.3%
Parkinson’s disease	Non-communicable	3	1.2%	1.1%	1.4%
Major depressive disorder	Non-communicable	3	3.7%	4.4%	2.9%
Diabetes mellitus	Non-communicable	3	22.0%	21.3%	22.8%
Chronic kidney disease	Non-communicable	3	40.4%	45.7%	33.7%
Skin and subcutaneous diseases	Non-communicable	3	52.3%	52.7%	51.9%
Other sense organ diseases ^2^	Non-communicable	3	6.0%	6.5%	5.4%
Rheumatoid arthritis	Non-communicable	3	1.0%	1.3%	0.6%
Osteoarthritis	Non-communicable	3	22.4%	25.4%	18.4%
Low back pain	Non-communicable	3	19.1%	20.7%	17.0%
Gout	Non-communicable	3	2.8%	1.7%	4.2%
Urinary system diseases ^3^	Non-communicable	3	5.6%	0.2%	12.5%
Genital prolapse	Non-communicable	4	4.5%	8.0%	0%
Endocrine, metabolic, blood, and immune disorders ^4^	Non-communicable	3	2.4%	2.4%	2.4%
Edentulism and severe tooth loss	Non-communicable	4	23.5%	27.1%	18.9%
Falls (injurious)	Injury	3	21.8%	22.8%	20.5%
Low physical activity	Risk factor	3	1.0%	0.9%	1.0%
High LDL cholesterol	Risk factor	3	35.0%	38.4%	30.7%
High systolic blood pressure	Risk factor	3	32.2%	34.2%	29.7%
Low bone mineral density	Risk factor	3	22.7%	27.1%	16.9%
Hearing loss	Impairment	1	74.8%	74.0%	75.8%
Heart failure	Impairment	1	8.8%	9.0%	8.6%
Blindness and vision impairment	Impairment	1	63.6%	65.0%	61.9%
**Mean GBD-FI score**	**Mixed**	**Mixed**	**0.155**	**0.161**	**0.148**

Prevalence values are coloured from lowest (dark green) to highest (dark red) as follows: dark green, light green, yellow, orange, and light red, and dark red. ^1^ A residual GBD cause group composed of cardiovascular diseases other than rheumatic heart disease, ischemic heart disease; stroke; hypertensive heart disease; non-rheumatic valvular heart disease; cardiomyopathy and myocarditis; atrial fibrillation and flutter; aortic aneurysm; peripheral artery disease and endocarditis [24]. ^2^ A residual GBD cause group including a plethora of eye and ear disorders such as disorders of the eyelids and vertiginous syndromes [24]. ^3^ This cause is “urinary diseases and male infertility” including urinary tract infections, urolithiasis, benign prostatic hyperplasia, male infertility and other urinary diseases but the GBD estimates zero prevalence for male infertility from age 50, so male infertility does not feature in these estimates. ^4^ “Endocrine, metabolic, blood, and immune disorders” is a residual GBD cause group, which includes mainly thyroid disorders, metabolic and immune disorders, and blood disorders, not including anaemia, diabetes, obesity and hypercholesterolemia [24]. ^5^ GBD level refers to an item’s hierarchical position in the GBD 2017 study, e.g., "Non-communicable diseases" (level 1), "Neoplasms" (level 2), "Liver cancer" (level 3), "Liver cancer due to hepatitis B" (level 4).

**Table 2:** Global prevalence (%) of individual deficits (*n* = 36) included in the GBD-FI for those aged ≥70 in 2017, compared by four world regions and the countries with the lowest and highest GBD-FI scores and the United Kingdom.

GBD-FI Items (*n* = 36)	Asia	Americas	Africa	Europe	China	Hungary	UK
Diarrheal diseases (from enteric infections)	2.0%	2.3%	2.7%	0.9%	1.1%	2.7%	1.5%
Protein-energy malnutrition	1.3%	1.4%	1.0%	0.8%	1.9%	0.1%	0.6%
Neoplasms	4.0%	12.9%	2.1%	8.0%	4.1%	6.0%	12.7%
Ischemic heart disease	10.7%	12.5%	13.3%	15.8%	9.2%	21.1%	15.7%
Stroke	8.5%	8.2%	6.8%	8.5%	11.3%	11.4%	6.4%
Non-rheumatic valvular heart disease	2.2%	5.7%	1.7%	5.9%	1.3%	6.1%	6.7%
Cardiomyopathy and myocarditis	0.3%	0.8%	0.7%	1.0%	0.1%	1.6%	0.4%
Atrial fibrillation and flutter	3.9%	8.0%	3.4%	6.3%	4.2%	6.2%	7.7%
Peripheral artery disease	10.7%	15.2%	11.9%	12.6%	11.0%	12.4%	12.3%
Other cardiovascular and circulatory diseases ^1^	3.5%	5.5%	5.9%	8.1%	1.6%	9.3%	8.3%
Chronic respiratory diseases	25.6%	27.1%	21.8%	27.5%	24.3%	38.2%	33.4%
Peptic ulcer disease	0.7%	0.7%	1.0%	0.7%	0.6%	1.2%	0.4%
Gallbladder and biliary diseases	0.9%	1.2%	0.5%	1.6%	1.2%	2.2%	1.2%
Alzheimer’s disease and other dementias	8.3%	8.1%	7.2%	10.2%	9.0%	10.1%	8.9%
Parkinson’s disease	1.2%	1.2%	1.0%	1.3%	1.4%	1.4%	1.2%
Major depressive disorder	4.0%	2.5%	5.5%	3.6%	4.2%	4.4%	3.0%
Diabetes mellitus	18.8%	24.8%	27.5%	26.1%	13.4%	28.8%	27.1%
Chronic kidney disease	35.9%	47.8%	52.4%	43.0%	26.4%	43.3%	34.9%
Skin and subcutaneous diseases	50.7%	51.3%	54.0%	56.7%	51.1%	53.4%	59.8%
Other sense organ diseases ^2^	6.0%	6.1%	5.8%	6.2%	6.0%	6.3%	6.1%
Rheumatoid arthritis	0.9%	1.2%	0.8%	1.1%	0.8%	0.8%	1.7%
Osteoarthritis	19.9%	29.6%	19.1%	24.0%	15.2%	20.1%	27.4%
Low back pain	15.4%	21.2%	22.6%	25.8%	9.8%	31.4%	24.3%
Gout	2.5%	3.3%	2.7%	3.0%	2.1%	2.1%	4.2%
Urinary system diseases ^3^	5.1%	5.1%	9.7%	6.2%	4.7%	5.4%	5.1%
Genital prolapse	4.0%	4.7%	8.6%	4.5%	3.2%	4.4%	5.9%
Endocrine, metabolic, blood, and immune disorders ^4^	2.2%	3.3%	2.3%	2.4%	1.6%	2.1%	2.5%
Edentulism and severe tooth loss	19.9%	30.6%	16.2%	29.0%	20.1%	31.1%	25.6%
Falls (injurious)	14.3%	25.3%	11.7%	39.9%	10.3%	81.0%	29.7%
Low physical activity	0.9%	1.0%	0.9%	1.1%	0.8%	0.8%	1.1%
High LDL cholesterol	32.6%	35.9%	26.9%	42.1%	33.3%	39.0%	42.2%
High systolic blood pressure	31.7%	27.3%	37.1%	35.8%	31.5%	58.6%	28.2%
Low bone mineral density	25.2%	19.5%	25.9%	18.0%	26.3%	18.0%	17.0%
Hearing loss	75.9%	73.8%	74.7%	72.9%	76.4%	75.4%	68.9%
Heart failure	8.3%	9.9%	8.1%	9.5%	8.1%	13.3%	8.0%
Blindness and vision impairment	77.1%	44.4%	85.9%	39.9%	71.3%	51.9%	22.2%
**Mean GBD-FI score**	**0.149**	**0.161**	**0.161**	**0.167**	**0.139**	**0.195**	**0.156**

Prevalence values are coloured from lowest (dark green) to highest (dark red) as follows: dark green, light green, yellow, orange, and light red, and dark red. ^1^ A residual GBD cause group composed of cardiovascular diseases other than rheumatic heart disease, ischemic heart disease; stroke; hypertensive heart disease; non-rheumatic valvular heart disease; cardiomyopathy and myocarditis; atrial fibrillation and flutter; aortic aneurysm; peripheral artery disease and endocarditis [24]. ^2^ A residual GBD cause group including a plethora of eye and ear disorders such as disorders of the eyelids and vertiginous syndromes [24]. ^3^ This cause is “urinary diseases and male infertility” including urinary tract infections, urolithiasis, benign prostatic hyperplasia, male infertility and other urinary diseases but the GBD estimates zero prevalence for male infertility from age 50, so male infertility does not feature in these estimates. ^4^ “Endocrine, metabolic, blood, and immune disorders” is a residual GBD cause group, which includes mainly thyroid disorders, metabolic and immune disorders, and blood disorders, not including anaemia, diabetes, obesity and hypercholesterolemia [24].

**Table 3:** Goodness of fit of models (adjusted r^2^ values) for predicting mortality from non-communicable diseases at the country level (*n* = 195), adjusting for different combinations of Age (population proportion aged ≥85), Sex (population proportion female), SDI index (measure of development), and HAQ index (measure of health care function) and mean GBD-FI score. The absolute proportional change (%) in r^2^ caused by adding mean GBD-FI score (improvement in fit/prediction of model) is provided.

Country-Level One Year Mortality from Non-Communicable Diseases in 2017 per 100,000		Age	Sex	SDI	HAQ	SDI HAQ	SDI HAQ Age	SDI HAQ Sex	SDI HAQ Age Sex
**Model adj r^2^**	-	14.1%	6.8%	5.6%	11.1%	16.9%	18.5%	24.0%	27.0%
**Model + GBD-FI adj r^2^**	14.3%	30.7%	17.1%	26.3%	32.4%	35.2%	35.4%	38.6%	39.6%
**Improvement in adj r^2^**	+14.3%	+16.6%	+10.3%	+20.7%	+21.3%	+18.3%	+16.9%	+14.6%	+12.6%
***p*-value for r^2^ difference**	*p* < 0.001	*p* < 0.001	*p* < 0.001	*p* < 0.001	*p* < 0.001	*p* < 0.001	*p* < 0.001	*p* < 0.001	*p* < 0.001

Variables were added into the models in two blocks, with the second block being the addition of mean GBD-FI score Variable ranges: non-communicable deaths 2851–10390 per 100,000; proportion aged ≥85 4%–23%; proportion female 36%–71%; SDI 0.19–0.92; HAQ index 19–97; and mean GBD-FI score 0.14–0.19.

## Supplementary Materials

The following are available online at https://www.mdpi.com/1660-4601/17/16/5695/s1, Table S1: All GBD items (*n* = 554) considered for inclusion in the GBD-FI with Spearman’s correlation for age (r), the maximum global prevalence for 5-year age groups between 25 and ≥95 years (Max Prev) and the prevalence for those aged ≥70 years (≥70 Prev) in 2017, Table S2: Detailed overview of item selection for the GBD-FI with reasons for the exclusion of items (from 554 to 36), Table S3: Lists and categorisation of the items in the GBD-FI and 10 other frailty indexes, Figure S1: Visual presentation of the items in (a) the Global Burden of Disease frailty index (GBD-FI) and (b) the electronic frailty index (eFI).
