# Supplementary material for: Assessing Global Frailty Scores: Development of a Global Burden of Disease-Frailty Index (GBD-FI)
Source: Int J Environ Res Public Health. 2020 Aug 6;17(16):5695. doi: 10.3390/ijerph17165695 (PMC7460080; doi:10.3390/ijerph17165695)
Supplement: Supplementary file 1 [file ijerph-17-05695-s001.pdf]

Table S1. All GBD items (n=554) considered for inclusion in the GBD-FI with Spearman's correlation for age (r), the maximum global prevalence for 5-year age groups between 25 and ≥95 years (Max Prev) and the prevalence for those aged ≥70 years (≥70 Prev) in 2017.

Cause	GBD Code	Age r	Max Prev	≥70 Prev	Age	Prev	Met criteria	Selected	Reason
**Causes (n=359)** Permalink for selected items: http://ghdx.healthdata.org/gbd-results-tool?params=gbd-api-2017-permalink/425a1ed5486547e39a9772ad30dc8651									
**Communicable, maternal, neonatal, and nutritional diseases**	**A**	-1.0	68.5%	53.7%	0	1	X		Excluded Lvl1
HIV/AIDS and sexually transmitted infections	A.1	-0.8	27.2%	18.7%	0	1	X		
HIV/AIDS	A.1.1	-1.0	1.1%	0.2%	0	1	X		
HIV/AIDS - Drug-susceptible Tuberculosis	A.1.1.1	-1.0	<0.1%	<0.1%	0	0	X		
HIV/AIDS - Multidrug-resistant Tuberculosis without extensive drug resistance	A.1.1.2	-1.0	<0.1%	<0.1%	0	0	X		
HIV/AIDS - Extensively drug-resistant Tuberculosis	A.1.1.3	-1.0	<0.1%	<0.1%	0	0	X		
HIV/AIDS resulting in other diseases	A.1.1.4	-1.0	1.1%	0.2%	0	1	X		
Sexually transmitted infections excluding HIV	A.1.2	-0.8	26.7%	18.6%	0	1	X		
Syphilis	A.1.2.1	-1.0	1.1%	<0.1%	0	1	X		
Chlamydial infection	A.1.2.2	-1.0	3.6%	<0.1%	0	1	X		
Gonococcal infection	A.1.2.3	-1.0	1.6%	<0.1%	0	1	X		
Trichomoniasis	A.1.2.4	-1.0	3.7%	0.8%	0	1	X		
Genital herpes	A.1.2.5	-0.3	22.6%	18%	0	1	X		
Other sexually transmitted infections	A.1.2.6	-0.9	0.8%	0%	0	0	X		
Respiratory infections and tuberculosis	A.2	-0.8	35.3%	25.8%	0	1	X		
Tuberculosis	A.2.1	-0.7	32.8%	23.8%	0	1	X		
Latent tuberculosis infection	A.2.1.1	-0.8	32.7%	23.5%	0	1	X		
Drug-susceptible tuberculosis	A.2.1.2	0.7	0.4%	0.4%	0	0	X		
Multidrug-resistant tuberculosis without extensive drug resistance	A.2.1.3	0.6	<0.1%	<0.1%	0	0	X		
Extensively drug-resistant tuberculosis	A.2.1.4	0.8	<0.1%	<0.1%	1	0	X		
Lower respiratory infections	A.2.2	0.6	0.4%	0.1%	0	0	X		
Upper respiratory infections	A.2.3	-0.9	3.2%	2.1%	0	1	X		
Otitis media	A.2.4	-0.6	1%	0.5%	0	1	X		
Enteric infections	A.3	0.8	2.7%	1.8%	1	1	✓	X	Chose lower
Diarrheal diseases	A.3.1	0.8	2.7%	1.8%	1	1	✓	✓	Included
Typhoid and paratyphoid	A.3.2	-1.0	<0.1%	<0.1%	0	0	X		
Typhoid fever	A.3.2.1	-0.9	<0.1%	<0.1%	0	0	X		
Paratyphoid fever	A.3.2.2	-1.0	<0.1%	<0.1%	0	0	X		
Invasive Non-typhoidal Salmonella (iNTS)	A.3.3	-0.7	<0.1%	<0.1%	0	0	X		
Other intestinal infectious diseases	A.3.5	N/A	0%	0%	0	0	X		
Neglected tropical diseases and malaria	A.4	-1.0	19.3%	8.4%	0	1	X		
Malaria	A.4.1	-1.0	1.4%	0.4%	0	1	X		
Chagas disease	A.4.2	1.0	0.2%	0.2%	1	0	X		
Leishmaniasis	A.4.3	-0.5	<0.1%	<0.1%	0	0	X		
Visceral leishmaniasis	A.4.3.1	-1.0	<0.1%	<0.1%	0	0	X		
Cutaneous and mucocutaneous leishmaniasis	A.4.3.2	-0.5	<0.1%	<0.1%	0	0	X		
African trypanosomiasis	A.4.4	-1.0	<0.1%	<0.1%	0	0	X		
Schistosomiasis	A.4.5	-1.0	3%	1.4%	0	1	X		
Cysticercosis	A.4.6	0.9	0.2%	0.2%	1	0	X		
Cystic echinococcosis	A.4.7	-0.9	<0.1%	<0.1%	0	0	X		
Lymphatic filariasis	A.4.8	-1.0	1.1%	0.5%	0	1	X		
Onchocerciasis	A.4.9	-1.0	0.4%	0.1%	0	0	X		
Trachoma	A.4.10	1.0	0.5%	0.5%	1	0	X		
Dengue	A.4.11	-0.2	0.2%	<0.1%	0	0	X		
Yellow fever	A.4.12	-1.0	<0.1%	<0.1%	0	0	X		
Rabies	A.4.13	0.8	<0.1%	<0.1%	1	0	X		
Intestinal nematode infections	A.4.14	-1.0	13.5%	4.8%	0	1	X		
Ascariasis	A.4.14.1	-1.0	5.9%	2.1%	0	1	X		
Trichuriasis	A.4.14.2	-1.0	5.7%	1%	0	1	X		
Hookworm disease	A.4.14.3	-1.0	3%	1.9%	0	1	X		
Food-borne trematodiases	A.4.15	-0.9	1.8%	0.9%	0	1	X		

Leprosy	A.4.16	0.9	<0.1%	<0.1%	1	0	X		
Ebola	A.4.17	-0.3	<0.1%	<0.1%	0	0	X		
Zika virus	A.4.18	-0.9	<0.1%	<0.1%	0	0	X		
Guinea worm disease	A.4.19	-1.0	<0.1%	<0.1%	0	0	X		
Other neglected tropical diseases	A.4.20	-1.0	0.6%	0.2%	0	0	X		
Other infectious diseases	A.5	-1.0	1.3%	0.8%	0	1	X		
Meningitis	A.5.1	-0.9	0.1%	0.1%	0	0	X		
Pneumococcal meningitis	A.5.1.1	-0.5	<0.1%	<0.1%	0	0	X		
H influenzae type B meningitis	A.5.1.2	-1.0	<0.1%	<0.1%	0	0	X		
Meningococcal meningitis	A.5.1.3	-0.6	<0.1%	<0.1%	0	0	X		
Other meningitis	A.5.1.4	-1.0	<0.1%	<0.1%	0	0	X		
Encephalitis	A.5.2	0.8	0.1%	0.1%	1	0	X		
Diphtheria	A.5.3	-0.7	<0.1%	0%	0	0	X		
Whooping cough	A.5.4	-0.8	<0.1%	0%	0	0	X		
Tetanus	A.5.5	-1.0	<0.1%	<0.1%	0	0	X		
Measles	A.5.6	-0.9	<0.1%	0%	0	0	X		
Varicella and herpes zoster	A.5.7	1.0	0.2%	0.2%	1	0	X		
Acute hepatitis	A.5.8	-1.0	0.4%	0.2%	0	0	X		
Acute hepatitis A	A.5.8.1	-1.0	<0.1%	<0.1%	0	0	X		
Acute hepatitis B	A.5.8.2	-1.0	0.3%	0.1%	0	0	X		
Acute hepatitis C	A.5.8.3	0.9	<0.1%	<0.1%	1	0	X		
Acute hepatitis E	A.5.8.4	0.1	<0.1%	<0.1%	0	0	X		
Other unspecified infectious diseases	A.5.9	-1.0	0.7%	0.3%	0	0	X		
Maternal and neonatal disorders	A.6	-1.0	2.2%	0.7%	0	1	X		
Maternal disorders	A.6.1	-1.0	0.3%	<0.1%	0	0	X		
Maternal hemorrhage	A.6.1.1	-0.9	<0.1%	0%	0	0	X		
Maternal sepsis and other maternal infections	A.6.1.2	-0.9	<0.1%	0%	0	0	X		
Maternal hypertensive disorders	A.6.1.3	-0.9	0.1%	0%	0	0	X		
Maternal obstructed labor and uterine rupture	A.6.1.4	-1.0	<0.1%	<0.1%	0	0	X		
Maternal abortion and miscarriage	A.6.1.5	-0.9	<0.1%	0%	0	0	X		
Ectopic pregnancy	A.6.1.6	-0.9	<0.1%	0%	0	0	X		
Indirect maternal deaths	A.6.1.7	N/A	N/A	N/A	0	0	X		
Late maternal deaths	A.6.1.8	N/A	N/A	N/A	0	0	X		
Maternal deaths aggravated by HIV/AIDS	A.6.1.9	N/A	N/A	N/A	0	0	X		
Other maternal disorders	A.6.1.10	N/A	0%	0%	0	0	X		
Neonatal disorders	A.6.2	-1.0	1.9%	0.7%	0	1	X		
Neonatal preterm birth	A.6.2.1	-1.0	1.1%	0.5%	0	1	X		
Neonatal encephalopathy due to birth asphyxia and trauma	A.6.2.2	-1.0	0.8%	0.3%	0	0	X		
Neonatal sepsis and other neonatal infections	A.6.2.3	-1.0	0.2%	<0.1%	0	0	X		
Hemolytic disease and other neonatal jaundice	A.6.2.4	-1.0	<0.1%	<0.1%	0	0	X		
Other neonatal disorders	A.6.2.5	N/A	0%	0%	0	0	X		
Nutritional deficiencies	A.7	-1.0	23.9%	17.1%	0	1	X		
Protein-energy malnutrition	A.7.1	1.0	1.6%	1.2%	1	1	✓	✓	Included
Iodine deficiency	A.7.2	-0.6	1.8%	1.7%	0	1	X		
Vitamin A deficiency	A.7.3	-1.0	9.5%	4.8%	0	1	X		
Dietary iron deficiency	A.7.4	-1.0	15.5%	11.4%	0	1	X		
Other nutritional deficiencies	A.7.5	N/A	0%	0%	0	0	X		
**Non-communicable diseases**	**B**	1.0	100%	100%	1	0	X		Excluded lvl1
Neoplasms	B.1	0.9	8.2%	6.3%	1	1	✓	✓	Included
Lip and oral cavity cancer	B.1.1	0.9	0.1%	<0.1%	1	0	X		
Nasopharynx cancer	B.1.2	-0.1	<0.1%	<0.1%	0	0	X		
Other pharynx cancer	B.1.3	0.4	<0.1%	<0.1%	0	0	X		
Esophageal cancer	B.1.4	0.8	<0.1%	<0.1%	1	0	X		
Stomach cancer	B.1.5	0.9	0.3%	0.2%	1	0	X		
Colon and rectum cancer	B.1.6	0.9	1.3%	0.8%	1	1	✓	X	Chose higher
Liver cancer	B.1.7	0.7	<0.1%	<0.1%	0	0	X		
Liver cancer due to hepatitis B	B.1.7.1	0.3	<0.1%	<0.1%	0	0	X		
Liver cancer due to hepatitis C	B.1.7.2	1.0	<0.1%	<0.1%	1	0	X		
Liver cancer due to alcohol use	B.1.7.3	0.7	<0.1%	<0.1%	0	0	X		
Liver cancer due to NASH	B.1.7.4	0.9	<0.1%	<0.1%	1	0	X		
Liver cancer due to other causes	B.1.7.5	0.6	<0.1%	<0.1%	0	0	X		
Gallbladder and biliary tract cancer	B.1.8	1.0	<0.1%	<0.1%	1	0	X		
Pancreatic cancer	B.1.9	1.0	<0.1%	<0.1%	1	0	X		
Larynx cancer	B.1.10	0.6	<0.1%	<0.1%	0	0	X		
Tracheal, bronchus, and lung cancer	B.1.11	0.8	0.3%	0.3%	1	0	X		
Malignant skin melanoma	B.1.12	0.9	0.2%	0.1%	1	0	X		

Non-melanoma skin cancer	B.1.13	1.0	1.4%	0.3%	1	1	✓	X	Chose higher
Non-melanoma skin cancer (squamous-cell carcinoma)	B.1.13.1	1.0	1.2%	0.3%	1	1	✓	X	Chose higher
Non-melanoma skin cancer (basal-cell carcinoma)	B.1.13.2	1.0	0.2%	<0.1%	1	0	X		
Breast cancer	B.1.14	1.0	1.4%	0.9%	1	1	✓	X	Chose higher
Cervical cancer	B.1.15	-0.6	0.1%	<0.1%	0	0	X		
Uterine cancer	B.1.16	0.6	0.2%	0.2%	0	0	X		
Ovarian cancer	B.1.17	0.7	<0.1%	<0.1%	1	0	X		
Prostate cancer	B.1.18	0.9	1.2%	1.1%	1	1	✓	X	Chose higher
Testicular cancer	B.1.19	-0.9	<0.1%	<0.1%	0	0	X		
Kidney cancer	B.1.20	0.6	0.1%	0.1%	0	0	X		
Bladder cancer	B.1.21	0.9	0.4%	0.2%	1	0	X		
Brain and nervous system cancer	B.1.22	0.5	<0.1%	<0.1%	0	0	X		
Thyroid cancer	B.1.23	0.3	<0.1%	<0.1%	0	0	X		
Mesothelioma	B.1.24	0.9	<0.1%	<0.1%	1	0	X		
Hodgkin lymphoma	B.1.25	-0.7	<0.1%	<0.1%	0	0	X		
Non-Hodgkin lymphoma	B.1.26	0.8	0.2%	0.2%	1	0	X		
Multiple myeloma	B.1.27	0.8	<0.1%	<0.1%	1	0	X		
Leukemia	B.1.28	0.9	0.2%	0.1%	1	0	X		
Acute lymphoid leukemia	B.1.28.1	0.5	<0.1%	<0.1%	0	0	X		
Chronic lymphoid leukemia	B.1.28.2	0.9	<0.1%	<0.1%	1	0	X		
Acute myeloid leukemia	B.1.28.3	0.9	<0.1%	<0.1%	1	0	X		
Chronic myeloid leukemia	B.1.28.4	1.0	<0.1%	<0.1%	1	0	X		
Other leukemia	B.1.28.5	0.9	<0.1%	<0.1%	1	0	X		
Other malignant neoplasms	B.1.29	0.8	0.4%	0.3%	1	0	X		
Other neoplasms	B.1.30	1.0	1.4%	1.3%	1	1	✓	X	Chose higher
Myelodysplastic, myeloproliferative, and other hematopoietic neoplasms	B.1.30.1	1.0	0.2%	0.1%	1	0	X		
Benign and in situ intestinal neoplasms	B.1.30.2	0.7	0.3%	0.3%	1	0	X		
Benign and in situ cervical and uterine neoplasms	B.1.30.3	-0.9	<0.1%	<0.1%	0	0	X		
Other benign and in situ neoplasms	B.1.30.4	1.0	1.1%	1%	1	1	✓	X	Chose higher
Cardiovascular diseases	B.2	1.0	50%	38.9%	1	1	✓	X	Chose lower
Rheumatic heart disease	B.2.1	-1.0	0.8%	0.2%	0	0	X		
Ischemic heart disease	B.2.2	1.0	14.9%	12.3%	1	1	✓	✓	Included
Stroke	B.2.3	1.0	12.6%	8.4%	1	1	✓	✓	Included
Ischemic stroke	B.2.3.1	1.0	12.4%	7.9%	1	1	✓	X	Chose higher
Intracerebral hemorrhage	B.2.3.2	0.6	0.9%	0.6%	0	0	X		
Subarachnoid hemorrhage	B.2.3.3	0.6	0.5%	0.3%	0	0	X		
Hypertensive heart disease	B.2.4	1.0	4.5%	2.1%	1	1	✓	X	Chose Risk
Non-rheumatic valvular heart disease	B.2.5	1.0	8.5%	3.6%	1	1	✓	✓	Included
Non-rheumatic calcific aortic valve disease	B.2.5.1	1.0	5.2%	1.8%	1	1	✓	X	Chose higher
Non-rheumatic degenerative mitral valve disease	B.2.5.2	1.0	3.7%	1.8%	1	1	✓	X	Chose higher
Other non-rheumatic valve diseases	B.2.5.3	1.0	<0.1%	<0.1%	1	0	X		
Cardiomyopathy and myocarditis	B.2.6	1.0	1.2%	0.6%	1	1	✓	✓	Included
Myocarditis	B.2.6.1	1.0	0.2%	<0.1%	1	0	X		
Alcoholic cardiomyopathy	B.2.6.2	0.4	<0.1%	<0.1%	0	0	X		
Other cardiomyopathy	B.2.6.3	1.0	1%	0.4%	1	0	X		
Atrial fibrillation and flutter	B.2.7	1.0	8.5%	5.1%	1	1	✓	✓	Included
Aortic aneurysm	B.2.8	N/A	N/A	N/A	0	0	X		
Peripheral artery disease	B.2.9	1.0	19.5%	12%	1	1	✓	✓	Included
Endocarditis	B.2.10	1.0	0.3%	<0.1%	1	0	X		
Other cardiovascular and circulatory diseases	B.2.11	0.8	5.7%	5%	1	1	✓	✓	Included
Chronic respiratory diseases	B.3	1.0	35.5%	26.1%	1	1	✓	✓	Included
Chronic obstructive pulmonary disease	B.3.1	1.0	34.9%	22.8%	1	1	✓	X	Chose higher
Pneumoconiosis	B.3.2	0.9	<0.1%	<0.1%	1	0	X		
Silicosis	B.3.2.1	0.9	<0.1%	<0.1%	1	0	X		
Asbestosis	B.3.2.2	1.0	<0.1%	<0.1%	1	0	X		
Coal workers pneumoconiosis	B.3.2.3	0.9	<0.1%	<0.1%	1	0	X		
Other pneumoconiosis	B.3.2.4	1.0	<0.1%	<0.1%	1	0	X		
Asthma	B.3.3	0.9	6.2%	6%	1	1	✓	X	Chose higher
Interstitial lung disease and pulmonary sarcoidosis	B.3.4	1.0	0.8%	0.6%	1	0	X		
Other chronic respiratory diseases	B.3.5	N/A	0%	0%	0	0	X		
Digestive diseases	B.4	0.4	50%	47.3%	0	1	X		
Cirrhosis and other chronic liver diseases	B.4.1	0.2	37.7%	34.9%	0	1	X		
Cirrhosis and other chronic liver diseases due to hepatitis B	B.4.1.1	-0.8	6.6%	5.6%	0	1	X		

Cirrhosis and other chronic liver diseases due to hepatitis C	B.4.1.2	1.0	2.6%	2.5%	1	1	✓	X	Plausibility
Cirrhosis and other chronic liver diseases due to alcohol use	B.4.1.3	-0.1	0.8%	0.5%	0	0	X		
Cirrhosis due to NASH	B.4.1.4	0.3	28.6%	26%	0	1	X		
Cirrhosis and other chronic liver diseases due to other causes	B.4.1.5	1.0	0.4%	0.3%	1	0	X		
Upper digestive system diseases	B.4.2	0.6	21.6%	20.6%	0	1	X		
Peptic ulcer disease	B.4.2.1	1.0	1.7%	0.7%	1	1	✓	✓	Included
Gastritis and duodenitis	B.4.2.2	0.4	3.6%	3.4%	0	1	X		
Gastroesophageal reflux disease	B.4.2.3	0.6	19.6%	18.5%	0	1	X		
Appendicitis	B.4.3	0.5	<0.1%	<0.1%	0	0	X		
Paralytic ileus and intestinal obstruction	B.4.4	1.0	<0.1%	<0.1%	1	0	X		
Inguinal, femoral, and abdominal hernia	B.4.5	0.8	0.9%	0.8%	1	0	X		
Inflammatory bowel disease	B.4.6	0.3	0.3%	0.2%	0	0	X		
Vascular intestinal disorders	B.4.7	1.0	<0.1%	<0.1%	1	0	X		
Gallbladder and biliary diseases	B.4.8	1.0	1.4%	1.1%	1	1	✓	✓	Included
Pancreatitis	B.4.9	1.0	0.5%	0.3%	1	0	X		
Other digestive diseases	B.4.10	N/A	0%	0%	0	0	X		
Neurological disorders	B.5	-0.3	58.7%	44.6%	0	1	X		
Alzheimer's disease and other dementias	B.5.1	1.0	36.7%	8.6%	1	1	✓	✓	Included
Parkinson's disease	B.5.2	1.0	1.8%	1.2%	1	1	✓	✓	Included
Epilepsy	B.5.3	0.9	0.7%	0.6%	1	0	X		
Multiple sclerosis	B.5.4	0.6	<0.1%	<0.1%	0	0	X		
Motor neuron disease	B.5.5	0.8	<0.1%	<0.1%	1	0	X		
Headache disorders	B.5.6	-0.9	53.9%	38.3%	0	1	X		
Migraine	B.5.6.1	-1.0	27.4%	9.4%	0	1	X		
Tension-type headache	B.5.6.2	-0.8	40.5%	32.9%	0	1	X		
Other neurological disorders	B.5.7	1.0	<0.1%	<0.1%	1	0	X		
Mental disorders	B.6	-0.7	15.3%	13.8%	0	1	X		
Schizophrenia	B.6.1	-0.9	0.5%	0.2%	0	0	X		
Depressive disorders	B.6.2	0.6	6.5%	6.2%	0	1	X		
Major depressive disorder	B.6.2.1	1.0	4.1%	3.7%	1	1	✓	✓	Included
Dysthymia	B.6.2.2	0.3	3.1%	2.7%	0	1	X		
Bipolar disorder	B.6.3	-0.9	0.8%	0.5%	0	0	X		
Anxiety disorders	B.6.4	-0.7	4.9%	4.1%	0	1	X		
Eating disorders	B.6.5	-0.8	0.5%	0%	0	0	X		
Anorexia nervosa	B.6.5.1	-0.8	<0.1%	0%	0	0	X		
Bulimia nervosa	B.6.5.2	-0.8	0.4%	0%	0	0	X		
Autism spectrum disorders	B.6.6	-1.0	0.4%	0.3%	0	0	X		
Attention-deficit/hyperactivity disorder	B.6.7	-1.0	1.2%	<0.1%	0	1	X		
Conduct disorder	B.6.8	N/A	0%	0%	0	0	X		
Idiopathic developmental intellectual disability	B.6.9	-1.0	1.4%	0.4%	0	1	X		
Other mental disorders	B.6.10	-0.1	3.3%	2.8%	0	1	X		
Substance use disorders	B.7	-1.0	4.7%	1.1%	0	1	X		
Alcohol use disorders	B.7.1	-1.0	2.6%	0.8%	0	1	X		
Drug use disorders	B.7.2	-0.9	2.3%	0.3%	0	1	X		
Opioid use disorders	B.7.2.1	-0.9	1.4%	0.2%	0	1	X		
Cocaine use disorders	B.7.2.2	-1.0	0.1%	<0.1%	0	0	X		
Amphetamine use disorders	B.7.2.3	-0.6	0.3%	<0.1%	0	0	X		
Cannabis use disorders	B.7.2.4	-1.0	0.5%	<0.1%	0	0	X		
Other drug use disorders	B.7.2.5	-0.8	<0.1%	<0.1%	0	0	X		
Diabetes and kidney diseases	B.8	1.0	62.7%	47.2%	1	1	✓	X	Chose lower
Diabetes mellitus	B.8.1	1.0	23.6%	22%	1	1	✓	✓	Included
Diabetes mellitus type 1	B.8.1.1	-0.8	0.2%	0.2%	0	0	X		
Diabetes mellitus type 2	B.8.1.2	1.0	23.4%	21.8%	1	1	✓	X	Chose higher
Chronic kidney disease	B.8.2	1.0	68.4%	40.4%	1	1	✓	✓	Included
Chronic kidney disease due to diabetes mellitus type 1	B.8.2.1	-0.6	<0.1%	<0.1%	0	0	X		
Chronic kidney disease due to diabetes mellitus type 2	B.8.2.2	0.9	7.9%	7.1%	1	1	✓	X	Chose higher
Chronic kidney disease due to hypertension	B.8.2.3	1.0	2.7%	1.4%	1	1	✓	X	Chose higher
Chronic kidney disease due to glomerulonephritis	B.8.2.4	1.0	6.2%	2.3%	1	1	✓	X	Chose higher
Chronic kidney disease due to other and unspecified causes	B.8.2.5	1.0	54.3%	29.6%	1	1	✓	X	Chose higher
Acute glomerulonephritis	B.8.3	1.0	<0.1%	<0.1%	1	0	X		
Skin and subcutaneous diseases	B.9	1.0	74.9%	52.3%	1	1	✓	✓	Included
Dermatitis	B.9.1	0.9	4.6%	4.1%	1	1	✓	X	Chose higher

Atopic dermatitis	B.9.1.1	0.8	2.5%	2.1%	1	1	✓	X	Chose higher
Contact dermatitis	B.9.1.2	1.0	1.9%	1.8%	1	1	✓	X	Chose higher
Seborrhoeic dermatitis	B.9.1.3	0.9	0.3%	0.3%	1	0	X		
Psoriasis	B.9.2	0.2	1.7%	1.5%	0	1	X		
Bacterial skin diseases	B.9.3	1.0	0.4%	0.2%	1	0	X		
Cellulitis	B.9.3.1	1.0	0.2%	<0.1%	1	0	X		
Pyoderma	B.9.3.2	1.0	0.3%	0.2%	1	0	X		
Scabies	B.9.4	-0.6	2.9%	1.5%	0	1	X		
Fungal skin diseases	B.9.5	1.0	61.8%	31%	1	1	✓	X	Chose higher
Viral skin diseases	B.9.6	-0.7	1%	0.7%	0	0	X		
Acne vulgaris	B.9.7	-1.0	1.9%	0.2%	0	1	X		
Alopecia areata	B.9.8	-1.0	0.4%	0.2%	0	0	X		
Pruritus	B.9.9	0.9	2.2%	2%	1	1	✓	X	Chose higher
Urticaria	B.9.10	-1.0	1%	0.6%	0	0	X		
Decubitus ulcer	B.9.11	1.0	0.8%	0.1%	1	0	X		
Other skin and subcutaneous diseases	B.9.12	1.0	27.1%	24.3%	1	1	✓	X	Chose higher
Sense organ diseases	B.10	1.0	85.6%	78.8%	1	1	✓	X	Chose lower
Blindness and vision impairment	B.10.1	1.0	55.9%	50.2%	1	1	✓	X	Chose impair
Glaucoma	B.10.1.1	1.0	0.9%	0.7%	1	0	X		
Cataract	B.10.1.2	1.0	23%	13.2%	1	1	✓	X	Chose higher
Age-related macular degeneration	B.10.1.3	1.0	1.7%	0.8%	1	1	✓	X	Chose higher
Refraction disorders	B.10.1.4	0.9	6.4%	6.2%	1	1	✓	X	Chose higher
Near vision loss	B.10.1.5	1.0	41.1%	39.5%	1	1	✓	X	Chose higher
Other vision loss	B.10.1.6	1.0	4.3%	2.2%	1	1	✓	X	Chose higher
Age-related and other hearing loss	B.10.2	1.0	96.1%	74%	1	1	✓	X	Chose impair
Other sense organ diseases	B.10.3	1.0	6.9%	6%	1	1	✓	✓	Included
Musculoskeletal disorders	B.11	1.0	49.5%	46.2%	1	1	✓	X	Chose lower
Rheumatoid arthritis	B.11.1	0.7	1%	1%	1	1	✓	✓	Included
Osteoarthritis	B.11.2	1.0	34%	22.4%	1	1	✓	✓	Included
Low back pain	B.11.3	1.0	21.1%	19.1%	1	1	✓	✓	Included
Neck pain	B.11.4	0.6	10.2%	9.6%	0	1	X		
Gout	B.11.5	1.0	3.1%	2.8%	1	1	✓	✓	Included
Other musculoskeletal disorders	B.11.6	-0.2	9.5%	7%	0	1	X		
Other non-communicable diseases	B.12	0.3	73%	72.3%	0	1	X		
Congenital birth defects	B.12.1	0.2	1.3%	1.3%	0	1	X		
Neural tube defects	B.12.1.1	-1.0	<0.1%	<0.1%	0	0	X		
Congenital heart anomalies	B.12.1.2	-0.5	<0.1%	<0.1%	0	0	X		
Orofacial clefts	B.12.1.3	-0.9	0.1%	0.1%	0	0	X		
Down syndrome	B.12.1.4	-0.9	<0.1%	0%	0	0	X		
Turner syndrome	B.12.1.5	-1.0	<0.1%	<0.1%	0	0	X		
Klinefelter syndrome	B.12.1.6	-1.0	<0.1%	<0.1%	0	0	X		
Other chromosomal abnormalities	B.12.1.7	-0.9	<0.1%	0%	0	0	X		
Congenital musculoskeletal and limb anomalies	B.12.1.8	0.7	0.6%	0.6%	1	0	X		
Urogenital congenital anomalies	B.12.1.9	-0.3	<0.1%	<0.1%	0	0	X		
Digestive congenital anomalies	B.12.1.10	1.0	0.4%	0.3%	1	0	X		
Other congenital birth defects	B.12.1.11	-1.0	0.4%	0.3%	0	0	X		
Urinary diseases and male infertility	B.12.2	0.8	5.8%	5.6%	1	1	✓	✓	Included
Urinary tract infections	B.12.2.1	0.3	0.2%	<0.1%	0	0	X		
Urolithiasis	B.12.2.2	0.7	0.1%	0.1%	0	0	X		
Benign prostatic hyperplasia	B.12.2.3	0.9	5.8%	5.6%	1	1	✓	X	Chose higher
Male infertility	B.12.2.4	-0.8	1.2%	0%	0	1	X		
Other urinary diseases	B.12.2.5	N/A	0%	0%	0	0	X		
Gynecological diseases	B.12.3	-0.9	23.5%	5.2%	0	1	X		
Uterine fibroids	B.12.3.1	-0.7	7.4%	0.8%	0	1	X		
Polycystic ovarian syndrome	B.12.3.2	-0.8	1.4%	0%	0	1	X		
Female infertility	B.12.3.3	-0.8	2.7%	0%	0	1	X		
Endometriosis	B.12.3.4	-0.8	1.7%	0%	0	1	X		
Genital prolapse	B.12.3.5	0.8	4.9%	4.5%	1	1	✓	✓	Included
Premenstrual syndrome	B.12.3.6	-0.8	15.3%	0%	0	1	X		
Other gynecological diseases	B.12.3.7	-0.8	2.1%	0%	0	1	X		
Hemoglobinopathies and hemolytic anemias	B.12.4	-1.0	26%	22.2%	0	1	X		
Thalassemias	B.12.4.1	-1.0	<0.1%	<0.1%	0	0	X		
Thalassemias trait	B.12.4.2	-0.8	4.4%	3.8%	0	1	X		
Sickle cell disorders	B.12.4.3	-1.0	<0.1%	<0.1%	0	0	X		
Sickle cell trait	B.12.4.4	-1.0	6.2%	4%	0	1	X		
G6PD deficiency	B.12.4.5	-1.0	4.8%	3.3%	0	1	X		

G6PD trait	B.12.4.6	0.1	13.5%	11.9%	0	1	X		
Other hemoglobinopathies and hemolytic anemias	B.12.4.7	0.2	1.5%	1.4%	0	1	X		
Endocrine, metabolic, blood, and immune disorders	B.12.5	1.0	3.7%	2.4%	1	1	✓	✓	Included
Oral disorders	B.12.6	0.6	59.7%	58.9%	0	1	X		
Caries of deciduous teeth	B.12.6.1	N/A	0%	0%	0	0	X		
Caries of permanent teeth	B.12.6.2	-1.0	38.5%	31.1%	0	1	X		
Periodontal diseases	B.12.6.3	0.3	26.7%	22.7%	0	1	X		
Edentulism and severe tooth loss	B.12.6.4	1.0	27.4%	23.5%	1	1	✓	✓	Included
Other oral disorders	B.12.6.5	-0.6	2.6%	2.1%	0	1	X		
Sudden infant death syndrome	B.12.7	N/A	N/A	N/A	0	0	X		
**Injuries**	**C**		83.8%	51.2%					Excluded lvl1
Transport injuries	C.1	1.0	11.1%	8.3%	1	1	✓	X	Plausibility
Road injuries	C.1.1	1.0	8%	6.6%	1	1	✓	X	Chose higher
Pedestrian road injuries	C.1.1.1	1.0	2.3%	1.8%	1	1	✓	X	Chose higher
Cyclist road injuries	C.1.1.2	1.0	1.5%	1.3%	1	1	✓	X	Chose higher
Motorcyclist road injuries	C.1.1.3	0.7	1.4%	1.4%	1	1	✓	X	Chose higher
Motor vehicle road injuries	C.1.1.4	1.0	2.6%	1.7%	1	1	✓	X	Chose higher
Other road injuries	C.1.1.5	1.0	0.5%	0.4%	1	0	X		
Other transport injuries	C.1.2	1.0	3%	1.7%	1	1	✓	X	Chose higher
Unintentional injuries	C.2	1.0	76.6%	39.3%	1	1	✓	X	Chose lower
Falls	C.2.1	1.0	56.6%	21.8%	1	1	✓	✓	Included
Drowning	C.2.2	1.0	0.1%	<0.1%	1	0	X		
Fire, heat, and hot substances	C.2.3	1.0	3.3%	2.8%	1	1	✓	X	Plausibility
Poisonings	C.2.4	1.0	0.2%	0.1%	1	0	X		
Poisoning by carbon monoxide	C.2.4.1	1.0	<0.1%	<0.1%	1	0	X	X	
Poisoning by other means	C.2.4.2	1.0	0.1%	<0.1%	1	0	X		
Exposure to mechanical forces	C.2.5	1.0	9.3%	6.9%	1	1	✓	X	Plausibility
Unintentional firearm injuries	C.2.5.1	1.0	0.3%	0.3%	1	0	X		
Other exposure to mechanical forces	C.2.5.2	1.0	9%	6.6%	1	1	✓	X	Chose higher
Adverse effects of medical treatment	C.2.6	0.8	0.1%	0.1%	1	0	X		
Animal contact	C.2.7	1.0	1.4%	1.2%	1	1	✓	X	Plausibility
Venomous animal contact	C.2.7.1	0.6	0.4%	0.4%	0	0	X		
Non-venomous animal contact	C.2.7.2	1.0	1.1%	0.8%	1	1	✓	X	Chose higher
Foreign body	C.2.8	1.0	1.1%	0.6%	1	1	✓	X	Plausibility
Pulmonary aspiration and foreign body in airway	C.2.8.1	1.0	0.2%	<0.1%	1	0	X		
Foreign body in eyes	C.2.8.2	1.0	0.2%	<0.1%	1	0	X		
Foreign body in other body part	C.2.8.3	1.0	0.8%	0.4%	1	0	X		
Environmental heat and cold exposure	C.2.9	1.0	1.8%	1.2%	1	1	✓	X	Plausibility
Exposure to forces of nature	C.2.10	-1.0	0.4%	0.2%	0	0	X		
Other unintentional injuries	C.2.11	1.0	9.8%	5%	1	1	✓	X	Plausibility
Self-harm and interpersonal violence	C.3	-1.0	7.1%	4.5%	0	1	X		
Self-harm	C.3.1	1.0	0.6%	0.4%	1	0	X		
Self-harm by firearm	C.3.1.1	1.0	<0.1%	<0.1%	1	0	X		
Self-harm by other specified means	C.3.1.2	1.0	0.6%	0.4%	1	0	X		
Interpersonal violence	C.3.2	-1.0	6.2%	3.7%	0	1	X		
Physical violence by firearm	C.3.2.1	1.0	0.1%	<0.1%	1	0	X		
Physical violence by sharp object	C.3.2.2	0.6	0.4%	0.4%	0	0	X		
Sexual violence	C.3.2.3	-1.0	5.6%	2%	0	1	X		
Physical violence by other means	C.3.2.4	0.9	1.3%	1.2%	1	1	✓	X	Plausibility
Conflict and terrorism	C.3.3	-0.8	1.1%	0.3%	0	1	X		
Executions and police conflict	C.3.4	-0.6	0.1%	<0.1%	0	0	X		
**Risk factors (n=84)**<br>Permalink for selected items: http://ghdx.healthdata.org/gbd-results-tool?params=gbd-api-2017-permalink/e41438ada0265c5844800a38db6e9adf									
**All risk factors**	-	N/A	N/A	N/A	0	0	X		
Environmental and occupational risks	-	N/A	N/A	N/A	0	0	X		
Unsafe water, sanitation, and handwashing	-	N/A	N/A	N/A	0	0	X		
Unsafe water source	-	-1	33.5%	20.4%	0	1	X		
Unsafe sanitation	-	-1	29.8%	19.4%	0	1	X		
No access to handwashing facility	-	-1	32.2%	19.3%	0	1	X		
Air pollution	-	N/A	N/A	N/A	0	0	X		
Particulate matter pollution	-	N/A	N/A	N/A	0	0	X		
Ambient particulate matter pollution	-	-0.2	46.6%	45.4%	0	1	X		
Household air pollution from solid fuels	-	-1	26.5%	18.7%	0	1	X		
Ambient ozone pollution	-	0.5	79.2%	79%	0	1	X		
Other environmental risks	-	N/A	N/A	N/A	0	0	X		
Residential radon	-	-0.8	33.6%	32.7%	0	1	X		

Lead exposure	-	0.8	34.6%	34.1%	1	1	✓	X	Plausibility
Occupational risks	-	N/A	N/A	N/A	0	0	X		
Occupational carcinogens	-	N/A	N/A	N/A	0	0	X		
Occupational exposure to asbestos	-	1	21.7%	14.4%	1	1	✓	X	Plausibility
Occupational exposure to arsenic	-	-0.7	0.9%	0.2%	0	0	X		
Occupational exposure to benzene	-	-1	2.2%	0.1%	0	1	X		
Occupational exposure to beryllium	-	-0.8	0.2%	<0.1%	0	0	X		
Occupational exposure to cadmium	-	-0.8	0.4%	<0.1%	0	0	X		
Occupational exposure to chromium	-	-0.8	0.9%	0.2%	0	0	X		
Occupational exposure to diesel engine exhaust	-	-0.8	4.4%	0.9%	0	1	X		
Occupational exposure to formaldehyde	-	-1	2.3%	0.1%	0	1	X		
Occupational exposure to nickel	-	-0.7	0.9%	0.2%	0	0	X		
Occupational exposure to polycyclic aromatic hydrocarbons	-	-0.8	1.8%	0.4%	0	1	X		
Occupational exposure to silica	-	-0.7	8.5%	2.1%	0	1	X		
Occupational exposure to sulfuric acid	-	-0.7	1.8%	0.4%	0	1	X		
Occupational exposure to trichloroethylene	-	-0.8	0.5%	0.1%	0	0	X		
Occupational asthmagens	-	-0.9	22.3%	2.6%	0	1	X		
Occupational particulate matter, gases, and fumes	-	0.1	15.8%	13.8%	0	1	X		
Occupational noise	-	0.3	18%	16.5%	0	1	X		
Occupational injuries	-	N/A	0%	0%	0	0	X		
Occupational ergonomic factors	-	-0.9	23.9%	2.6%	0	1	X		
Behavioural risks	-	N/A	N/A	N/A	0	0	X		
Child and maternal malnutrition	-	N/A	N/A	N/A	0	0	X		
Suboptimal breastfeeding	-	N/A	N/A	N/A	0	0	X		
Non-exclusive breastfeeding	-	N/A	N/A	0%	0	0	X		
Discontinued breastfeeding	-	N/A	0%	0%	0	0	X		
Child growth failure	-	N/A	N/A	N/A	0	0	X		
Child underweight	-	N/A	0%	0%	0	0	X		
Child wasting	-	N/A	0%	0%	0	0	X		
Child stunting	-	N/A	0%	0%	0	0	X		
Low birth weight for gestation	-	N/A	0%	0%	0	0	X		
Low birthweight for gestation	-	N/A	N/A	N/A	0	0	X		
Short gestation for birth weight	-	N/A	0%	0%	0	0	X		
Iron deficiency	-	-1	7.3%	0%	0	1	X		
Vitamin A deficiency	-	N/A	0%	0%	0	0	X		
Zinc deficiency	-	N/A	0%	0%	0	0	X		
Tobacco	-	N/A	N/A	N/A	0	0	X		
Smoking	-	0.1	15.9%	12.7%	0	1	X		
Chewing tobacco	-	-0.1	7.9%	7.0%	0	1	X		
Second-hand smoke	-	-0.9	37.4%	25.4%	0	1	X		
Alcohol use	-	-0.7	16%	11.3%	0	1	X		
Drug use	-	-0.9	1.7%	0.3%	0	1	X		
Dietary risks	-	N/A	N/A	N/A	0	0	X		
Diet low in fruits	-	-0.6	67.6%	55.7%	0	1	X		
Diet low in vegetables	-	0.6	60.8%	53%	0	1	X		
Diet low in legumes	-	1	57.1%	49.4%	1	1	✓	X	Plausibility
Diet low in whole grains	-	1	77.2%	75.1%	1	1	✓	X	Plausibility
Diet low in nuts and seeds	-	-0.1	87.4%	85.5%	0	1	X		
Diet low in milk	-	-1	85.3%	76.7%	0	1	X		
Diet high in red meat	-	-0.7	22.5%	14.7%	0	1	X		
Diet high in processed meat	-	0.7	9.7%	8.4%	1	1	✓	X	Plausibility
Diet high in sugar-sweetened beverages	-	-0.5	14.2%	7.6%	0	1	X		
Diet low in fibre	-	0.3	64.5%	59.3%	0	1	X		
Diet low in calcium	-	-0.7	71.8%	65.8%	0	1	X		
Diet low in seafood omega-3 fatty acids	-	-0.9	78.9%	71.8%	0	1	X		
Diet low in polyunsaturated fatty acids	-	-1	65.5%	57.4%	0	1	X		
Diet high in trans fatty acids	-	1	7.9%	6.4%	1	1	✓	X	Plausibility
Diet high in sodium	-	-0.5	32.8%	24%	0	1	X		
Intimate partner violence	-	-0.9	7.9%	0%	0	1	X		
Childhood maltreatment	-	N/A	N/A	N/A	0	0	X		
Childhood sexual abuse	-	-1	10.9%	5.5%	0	1	X		
Bullying victimization	-	-0.7	7%	0%	0	1	X		
Unsafe sex	-	N/A	0%	0%	0	0	X		
Low physical activity	-	1	1%	1%	1	1	✓	✓	Included
Metabolic risks	-	N/A	N/A	N/A	0	0			
High fasting plasma glucose	-	0.8	22%	15.2%	1	1	✓	X	Chose cause

High LDL cholesterol	-	0.9	35.8%	35%	1	1	✓	✓	Included
High systolic blood pressure	-	0.9	35.6%	32.2%	1	1	✓	✓	Included
High body-mass index	-	1	16.5%	12.3%	1	1	✓	X	Plausibility
Low bone mineral density	-	0.9	26.3%	22.7%	1	1	✓	✓	Included
Impaired kidney function	-	0.9	19.4%	11.2%	1	1	✓	✓	Chose cause
**Impairments (n=40)**<br>Permalink for selected items: http://ghdx.healthdata.org/gbd-results-tool?params=gbd-api-2017-permalink/d28f66de23ee6fd6aa46b906f7a581cf									
Anemia	-	0	24.9%	23.9%	0	1	X		
Mild anemia	-	0.2	16.1%	15.5%	0	1	X		
Moderate anemia	-	-0.3	8.2%	7.9%	0	1	X		
Severe anemia	-	0	0.6%	0.6%	0	0	X		
Epilepsy	-	0.7	0.9%	0.9%	0	0	X		
Treated epilepsy	-	0.9	0.3%	0.3%	1	0	X		
Moderate epilepsy	-	-0.3	0.3%	0.3%	0	0	X		
Severe epilepsy	-	0.1	0.4%	0.3%	0	0	X		
Guillain-Barré syndrome	-	1	<0.1%	<0.1%	1	0	X		
Hearing loss	-	1	96.9%	74.8%	1	1	✓	✓	Included
Mild hearing loss	-	0.8	45.7%	43%	1	1	✓	X	Chose higher
Moderate hearing loss	-	1	32.6%	19.2%	1	1	✓	X	Chose higher
Moderately severe hearing loss	-	1	24.8%	10.1%	1	1	✓	X	Chose higher
Severe hearing loss	-	1	4.2%	1.4%	1	1	✓	X	Chose higher
Profound hearing loss	-	0.8	1.8%	0.6%	1	1	✓	X	Chose higher
Complete hearing loss	-	1	1.2%	0.4%	1	1	✓	X	Chose higher
Heart failure	-	1	20.6%	8.8%	1	1	✓	✓	Included
Mild heart failure	-	1	3.8%	1.6%	1	1	✓	X	Chose higher
Moderate heart failure	-	1	2.5%	1.1%	1	1	✓	X	Chose higher
Severe heart failure	-	1	6.7%	2.9%	1	1	✓	X	Chose higher
Treated heart failure	-	1	7.6%	3.3%	1	1	✓	X	Chose higher
Infertility	-	-1	5.2%	<0.1%	0	1	X		
Primary infertility	-	-1	1.2%	<0.1%	0	1	X		
Secondary infertility	-	-0.8	4.4%	0%	0	1	X		
Developmental intellectual disability	-	-1	2.7%	0.9%	0	1	X		
Borderline intellectual disability	-	-1	0.5%	0.2%	0	0	X		
Mild intellectual disability	-	-1	1.4%	0.4%	0	1	X		
Moderate intellectual disability	-	-1	0.4%	0.1%	0	0	X		
Severe intellectual disability	-	-1	0.3%	<0.1%	0	0	X		
Profound intellectual disability	-	-1	<0.1%	<0.1%	0	0	X		
Pelvic inflammatory disease	-	-0.9	<0.1%	0%	0	0	X		
Moderate pelvic inflammatory disease	-	-0.9	<0.1%	0%	0	0	X		
Severe pelvic inflammatory disease	-	-0.9	<0.1%	0%	0	0	X		
Blindness and vision impairment	-	1	76.5%	63.6%	1	1	✓	✓	Included
Mild vision loss	-	-1	<0.1%	<0.1%	0	0	X		
Moderate vision loss	-	1	21.9%	16.4%	1	1	✓	X	Chose higher
Severe vision loss	-	1	4.6%	3.3%	1	1	✓	X	Chose higher
Blindness	-	1	9.9%	4.4%	1	1	✓	X	Chose higher
Monocular vision loss	-	0.9	<0.1%	<0.1%	1	0	X		
Presbyopia	-	1	41.1%	39.5%	1	1	✓	X	Chose higher
**Aetiologies (n=17)**									
Cholera	-	N/A	N/A	N/A	0	0	X		
Non-typhoidal Salmonella	-	N/A	N/A	N/A	0	0	X		
Shigella	-	N/A	N/A	N/A	0	0	X		
Enteropathogenic E coli	-	N/A	N/A	N/A	0	0	X		
Enterotoxigenic E coli	-	N/A	N/A	N/A	0	0	X		
Campylobacter	-	N/A	N/A	N/A	0	0	X		
Entamoeba	-	N/A	N/A	N/A	0	0	X		
Cryptosporidium	-	N/A	N/A	N/A	0	0	X		
Rotavirus	-	N/A	N/A	N/A	0	0	X		
Aeromonas	-	N/A	N/A	N/A	0	0	X		
Clostridium difficile	-	N/A	N/A	N/A	0	0	X		
Norovirus	-	N/A	N/A	N/A	0	0	X		
Adenovirus	-	N/A	N/A	N/A	0	0	X		
Influenza	-	N/A	N/A	N/A	0	0	X		
Pneumococcal pneumonia	-	N/A	N/A	N/A	0	0	X		
H influenza type B	-	N/A	N/A	N/A	0	0	X		
Respiratory syncytial virus	-	N/A	N/A	N/A	0	0	X		
**Injuries by nature (n=54)**									

Amputations	-	1	20.1%	13%	1	1	✓	X	Not included
Amputation of lower limbs, bilateral	-	1	0.6%	0.3%	1	0	X		
Amputation of upper limbs, bilateral	-	1	0.7%	0.4%	1	0	X		
Amputation of fingers (excluding thumb)	-	1	7.0%	5.4%	1	1	✓	X	Not included
Amputation of lower limb, unilateral	-	1	3.0%	1.4%	1	1	✓	X	Not included
Amputation of upper limb, unilateral	-	1	0.8%	0.4%	1	0	X		
Amputation of thumb	-	1	4.1%	2.7%	1	1	✓	X	Not included
Amputation of toe/toes	-	1	3.9%	2.4%	1	1	✓	X	Not included
Burns	-	1	7.7%	5.9%	1	1	✓	X	Not included
Lower airway burns	-	1	<0.1%	<0.1%	1	0	X		
Burns, <20% total burned surface area without lower airway burns	-	1	7.7%	5.8%	1	1	✓	X	Not included
Burns, >=20% total burned surface area or >= 10% burned surface area if head/neck or hands/wrist involved w/o lower airway burns	-	-0.9	0.3%	<0.1%	0	0	X		
Fractures	-	1	49.4%	19.6%	1	1	✓	X	Not included
Fracture of clavicle, scapula, or humerus	-	1	0.8%	0.3%	1	0	X		
Fracture of face bones	-	1	0.1%	<0.1%	1	0	X		
Fracture of foot bones except ankle	-	1	1.5%	0.8%	1	1	✓	X	Not included
Fracture of hand (wrist and other distal part of hand)	-	1	2.1%	1.1%	1	1	✓	X	Not included
Fracture of hip	-	1	4.9%	1.5%	1	1	✓	X	Not included
Fracture of patella, tibia or fibula, or ankle	-	1	30.1%	12.3%	1	1	✓	X	Not included
Fracture of pelvis	-	1	1.2%	0.6%	1	1	✓	X	Not included
Fracture of radius and/or ulna	-	1	0.6%	0.2%	1	0	X		
Fracture of skull	-	1	0.3%	0.1%	1	0	X		
Fracture of sternum and/or fracture of one or more ribs	-	1	0.3%	<0.1%	1	0	X		
Fracture of vertebral column	-	1	1%	0.3%	1	1	✓	X	Not included
Fracture of femur, other than femoral neck	-	1	7.7%	2.3%	1	1	✓	X	Not included
Head Injuries	-	1	1.8%	1.5%	1	1	✓	X	Not included
Minor TBI	-	1	1.2%	0.4%	1	1	✓	X	Not included
Moderate/Severe TBI	-	0.5	1.1%	1.1%	0	1	X		
Spinal Injuries	-	0.2	0.6%	0.5%	0	0	X		
Spinal cord lesion at neck level	-	-0.2	0.3%	0.2%	0	0	X		
Spinal cord lesion below neck level	-	0.5	0.3%	0.3%	0	0	X		
Minor Injuries	-	1	14.7%	7.8%	1	1	✓	X	Not included
Muscle and tendon injuries, including sprains and strains lesser dislocations	-	1	3.9%	2.4%	1	1	✓	X	Not included
Foreign body in ear	-	0.4	<0.1%	<0.1%	0	0	X		
Open wound(s)	-	1	8.5%	4.4%	1	1	✓	X	Not included
Contusion in any part of the body	-	1	2.2%	0.9%	1	1	✓	X	Not included
Superficial injury of any part of the body	-	0.5	<0.1%	<0.1%	0	0	X		
Other Injuries	-	1	5.3%	2.5%	1	1	✓	X	Not included
Dislocation of hip	-	1	1.2%	0.5%	1	1	✓	X	Not included
Dislocation of knee	-	1	0.1%	<0.1%	1	0	X		
Dislocation of shoulder	-	1	<0.1%	<0.1%	1	0	X		
Foreign body in respiratory system	-	0.5	<0.1%	<0.1%	0	0	X		
Foreign body in GI and urogenital system	-	0.6	<0.1%	<0.1%	0	0	X		
Drowning and nonfatal submersion	-	1	<0.1%	<0.1%	1	0	X		
Asphyxiation	-	1	<0.1%	<0.1%	1	0	X		
Crush injury	-	1	0.5%	0.2%	1	0	X		
Nerve injury	-	1	0.6%	0.3%	1	0	X		
Injury to eyes	-	-0.2	<0.1%	<0.1%	0	0	X		
Poisoning requiring urgent care	-	0.9	0.2%	0.1%	1	0	X		
Severe chest Injury	-	1	0.2%	<0.1%	1	0	X		
Internal hemorrhage in abdomen and pelvis	-	1	0.5%	0.2%	1	0	X		
Effect of different environmental factors	-	1	1.3%	0.6%	1	1	✓	X	Not included
Complications following therapeutic procedures	-	1	0.2%	0.1%	1	0	X		
Multiple fractures, dislocations, crashes, wounds, pains, and strains	-	0	0.3%	0.2%	0	0	X		

Table S2. Detailed overview of item selection for the GBD-FI with reasons for the exclusion of items (from 554 to 36).

Unsuitable categories (n=74)

Level 1 causes (n=3)

Communicable, maternal, neonatal, and nutritional diseases; Non-communicable diseases; Injuries.

Aetiologies (n=17)

Cholera; Non-typhoidal Salmonella; Shigella; Enteropathogenic E coli; Enterotoxigenic E coli; Campylobacter; Entamoeba; Cryptosporidium; Rotavirus; Aeromonas; Clostridium difficile; Norovirus; Adenovirus; Influenza; Pneumococcal pneumonia; H influenza type B; Respiratory syncytial virus.

Injuries by nature (n=54)

Amputations; Amputation of lower limbs, bilateral; Amputation of upper limbs, bilateral; Amputation of fingers (excluding thumb); Amputation of lower limb, unilateral; Amputation of upper limb, unilateral; Amputation of thumb; Amputation of toe/toes; Burns; Lower airway burns; Burns, <20% total burned surface area without lower airway burns; Burns, >=20% total burned surface area or >= 10% burned surface area if head/neck or hands/wrist involved w/o lower airway burns; Fractures; Fracture of clavicle, scapula, or humerus; Fracture of face bones; Fracture of foot bones except ankle; Fracture of hand (wrist and other distal part of hand); Fracture of hip; Fracture of patella, tibia or fibula, or ankle; Fracture of pelvis; Fracture of radius and/or ulna; Fracture of skull; Fracture of sternum and/or fracture of one or more ribs; Fracture of vertebral column; Fracture of femur, other than femoral neck; Head Injuries; Minor TBI; Moderate/Severe TBI; Spinal Injuries; Spinal cord lesion at neck level; Spinal cord lesion below neck level; Minor Injuries; Muscle and tendon injuries, including sprains and strains lesser dislocations; Foreign body in ear; Open wound(s); Contusion in any part of the body; Superficial injury of any part of the body; Other Injuries; Dislocation of hip; Dislocation of knee; Dislocation of shoulder; Foreign body in respiratory system; Foreign body in GI and urogenital system; Drowning and nonfatal submersion; Asphyxiation; Crush injury; Nerve injury; Injury to eyes; Poisoning requiring urgent care; Severe chest Injury; Internal hemorrhage in abdomen and pelvis; Effect of different environmental factors; Complications following therapeutic procedures; Multiple fractures, dislocations, crashes, wounds, pains, and strains.

Missing prevalence data (n=42)

Other intestinal infectious diseases; Indirect maternal deaths; Late maternal deaths; Maternal deaths aggravated by HIV/AIDS; Other maternal disorders; Other neonatal disorders; Other nutritional deficiencies; Aortic aneurysm; Other chronic respiratory diseases; Other digestive diseases; Conduct disorder; Other urinary diseases; Caries of deciduous teeth; Sudden infant death syndrome; All risk factors; Environmental and occupational risks; Unsafe water, sanitation, and handwashing; Air pollution; Particulate matter pollution; Other environmental risks; Occupational risks; Occupational carcinogens; Occupational injuries; Behavioural risks; Child and maternal malnutrition; Suboptimal breastfeeding; Non-exclusive breastfeeding; Discontinued breastfeeding; Child growth failure; Child underweight; Child wasting; Child stunting; Low birth weight for gestation; Low birthweight for gestation; Short gestation for birth weight; Vitamin A deficiency; Zinc deficiency; Tobacco; Dietary risks; Childhood maltreatment; Unsafe sex; Metabolic risks.

Not strong age-correlation (r<0.7; n=248)

HIV/AIDS and sexually transmitted infections; HIV/AIDS; HIV/AIDS - Drug-susceptible Tuberculosis; HIV/AIDS - Multidrug-resistant Tuberculosis without extensive drug resistance; HIV/AIDS - Extensively drug-resistant Tuberculosis; HIV/AIDS resulting in other diseases; Sexually transmitted infections excluding HIV; Syphilis; Chlamydial infection; Gonococcal infection; Trichomoniasis; Genital herpes; Other sexually transmitted infections; Respiratory infections and tuberculosis; Tuberculosis; Latent tuberculosis infection; Drug-susceptible tuberculosis; Multidrug-resistant tuberculosis without extensive drug resistance; Lower respiratory infections; Upper respiratory infections; Otitis media; Typhoid and paratyphoid; Typhoid fever; Paratyphoid fever; Invasive Non-typhoidal Salmonella (iNTS); Neglected tropical diseases and malaria; Malaria; Leishmaniasis; Visceral leishmaniasis; Cutaneous and mucocutaneous leishmaniasis; African trypanosomiasis; Schistosomiasis; Cystic echinococcosis; Lymphatic filariasis; Onchocerciasis; Dengue; Yellow fever; Intestinal nematode infections; Ascariasis; Trichuriasis; Hookworm disease; Food-borne trematodiases; Ebola; Zika virus; Guinea worm disease; Other neglected tropical diseases; Other infectious diseases; Meningitis; Pneumococcal meningitis; H influenzae type B meningitis; Meningococcal meningitis; Other meningitis; Diphtheria; Whooping cough; Tetanus; Measles; Acute hepatitis; Acute hepatitis A; Acute hepatitis B; Acute hepatitis E; Other unspecified infectious diseases; Maternal and neonatal disorders; Maternal disorders; Maternal hemorrhage; Maternal sepsis and other maternal infections; Maternal hypertensive disorders; Maternal obstructed labor and uterine rupture; Maternal abortion and miscarriage; Ectopic pregnancy; Neonatal disorders; Neonatal preterm birth; Neonatal encephalopathy due to birth asphyxia and trauma; Neonatal sepsis and other neonatal infections; Hemolytic disease and other neonatal jaundice; Nutritional deficiencies; Iodine deficiency; Vitamin A deficiency; Dietary iron deficiency; Nasopharynx cancer; Other pharynx cancer; Liver cancer; Liver cancer due to hepatitis B; Liver cancer due to alcohol use; Liver cancer due to other causes; Larynx cancer; Cervical cancer; Uterine cancer; Testicular cancer; Kidney cancer; Brain and nervous system cancer; Thyroid cancer; Hodgkin lymphoma; Acute lymphoid leukemia; Benign and in situ cervical and uterine neoplasms; Rheumatic heart disease; Intracerebral hemorrhage; Subarachnoid hemorrhage; Alcoholic cardiomyopathy; Digestive diseases; Cirrhosis and other chronic liver diseases; Cirrhosis and other chronic liver diseases due to hepatitis B; Cirrhosis and other chronic liver diseases due to alcohol use; Cirrhosis due to NASH; Upper digestive system diseases; Gastritis and duodenitis; Gastroesophageal reflux disease; Appendicitis; Inflammatory bowel disease; Neurological disorders; Multiple sclerosis; Headache disorders; Migraine; Tension-type headache; Mental disorders; Schizophrenia; Depressive disorders; Dysthymia; Bipolar disorder; Anxiety disorders; Eating disorders; Anorexia nervosa; Bulimia nervosa; Autism spectrum disorders; Attention-deficit/hyperactivity disorder; Idiopathic developmental intellectual disability; Other mental disorders; Substance use disorders; Alcohol use disorders; Drug use disorders; Opioid use disorders; Cocaine use disorders; Amphetamine use disorders; Cannabis use disorders; Other drug use disorders; Diabetes mellitus type 1; Chronic kidney disease due to diabetes mellitus type 1; Psoriasis; Scabies; Viral skin diseases; Acne vulgaris; Alopecia areata; Urticaria; Neck pain; Other musculoskeletal disorders; Other non-communicable diseases; Congenital birth defects; Neural tube defects; Congenital heart anomalies; Orofacial clefts; Down syndrome; Turner syndrome; Klinefelter syndrome; Other chromosomal abnormalities; Urogenital congenital anomalies; Other congenital birth defects; Urinary tract infections; Urolithiasis; Male infertility; Gynecological diseases; Uterine fibroids; Polycystic ovarian syndrome; Female infertility; Endometriosis; Premenstrual syndrome; Other gynecological diseases; Hemoglobinopathies and hemolytic anemias; Thalassemias; Thalassemias trait; Sickle cell disorders; Sickle cell trait; G6PD deficiency; G6PD trait; Other hemoglobinopathies and hemolytic anemias; Oral disorders; Caries of permanent teeth; Periodontal diseases; Other oral disorders; Venomous animal contact;

Exposure to forces of nature; Self-harm and interpersonal violence; Interpersonal violence; Physical violence by sharp object; Sexual violence; Conflict and terrorism; Executions and police conflict; Unsafe water source; Unsafe sanitation; No access to handwashing facility; Ambient particulate matter pollution; Household air pollution from solid fuels; Ambient ozone pollution; Residential radon; Occupational exposure to arsenic; Occupational exposure to benzene; Occupational exposure to beryllium; Occupational exposure to cadmium; Occupational exposure to chromium; Occupational exposure to diesel engine exhaust; Occupational exposure to formaldehyde; Occupational exposure to nickel; Occupational exposure to polycyclic aromatic hydrocarbons; Occupational exposure to silica; Occupational exposure to sulfuric acid; Occupational exposure to trichloroethylene; Occupational asthmagens; Occupational particulate matter, gases and fumes; Occupational noise; Occupational ergonomic factors; Iron deficiency; Smoking; Chewing tobacco; Second-hand smoke; Alcohol use; Drug use; Diet low in fruits; Diet low in vegetables; Diet low in nuts and seeds; Diet low in milk; Diet high in red meat; Diet high in sugar-sweetened beverages; Diet low in fibre; Diet low in calcium; Diet low in seafood omega-3 fatty acids; Diet low in polyunsaturated fatty acids; Diet high in sodium; Intimate partner violence; Childhood sexual abuse; Bullying victimization; Anemia; Mild anemia; Moderate anemia; Severe anemia; Epilepsy; Moderate epilepsy; Severe epilepsy; Infertility; Primary infertility; Secondary infertility; Developmental intellectual disability; Borderline intellectual disability; Mild intellectual disability; Moderate intellectual disability; Severe intellectual disability; Profound intellectual disability; Pelvic inflammatory disease; Moderate pelvic inflammatory disease; Severe pelvic inflammatory disease; Mild vision loss.
**Too rare (<1% prevalence; n=76)**<br>Extensively drug-resistant tuberculosis; Chagas disease; Cysticercosis; Trachoma; Rabies; Leprosy; Encephalitis; Varicella and herpes zoster; Acute hepatitis C; Lip and oral cavity cancer; Esophageal cancer; Stomach cancer; Liver cancer due to hepatitis C; Liver cancer due to NASH; Gallbladder and biliary tract cancer; Pancreatic cancer; Tracheal, bronchus, and lung cancer; Malignant skin melanoma; Non-melanoma skin cancer (basal-cell carcinoma); Ovarian cancer; Bladder cancer; Mesothelioma; Non-Hodgkin lymphoma; Multiple myeloma; Leukemia; Chronic lymphoid leukemia; Acute myeloid leukemia; Chronic myeloid leukemia; Other leukemia; Other malignant neoplasms; Myelodysplastic, myeloproliferative, and other hematopoietic neoplasms; Benign and in situ intestinal neoplasms; Other non-rheumatic valve diseases; Myocarditis; Other cardiomyopathy; Endocarditis; Pneumoconiosis; Silicosis; Asbestosis; Coal workers pneumoconiosis; Other pneumoconiosis; Interstitial lung disease and pulmonary sarcoidosis; Cirrhosis and other chronic liver diseases due to other causes; Paralytic ileus and intestinal obstruction; Inguinal, femoral, and abdominal hernia; Vascular intestinal disorders; Pancreatitis; Epilepsy; Motor neuron disease; Other neurological disorders; Acute glomerulonephritis; Seborrhoeic dermatitis; Bacterial skin diseases; Cellulitis; Pyoderma; Decubitus ulcer; Glaucoma; Congenital musculoskeletal and limb anomalies; Digestive congenital anomalies; Other road injuries; Drowning; Poisonings; Poisoning by carbon monoxide; Poisoning by other means; Unintentional firearm injuries; Adverse effects of medical treatment; Pulmonary aspiration and foreign body in airway; Foreign body in eyes; Foreign body in other body part; Self-harm; Self-harm by firearm; Self-harm by other specified means; Physical violence by firearm; Treated epilepsy; Guillain-Barré syndrome; Monocular vision loss.
**Duplication/redundancy (n=62)**<br>**Equivalent causes chosen (n=1)**<br>High fasting plasma glucose.<br>**Equivalent risk factors chosen (n=1)**<br>Hypertensive heart disease.<br>**Equivalent impairments chosen (n=2)**<br>Blindness and vision impairment; Age-related and other hearing loss.<br>**Higher order causes chosen (n=52)**<br>Colon and rectum cancer; Non-melanoma skin cancer; Non-melanoma skin cancer (squamous-cell carcinoma); Breast cancer; Prostate cancer; Other neoplasms; Other benign and in situ neoplasms; Ischemic stroke; Non-rheumatic calcific aortic valve disease; Non-rheumatic degenerative mitral valve disease; Chronic obstructive pulmonary disease; Asthma; Diabetes mellitus type 1; Diabetes mellitus type 2; Chronic kidney disease due to diabetes mellitus type 2; Chronic kidney disease due to hypertension; Chronic kidney disease due to glomerulonephritis; Chronic kidney disease due to other and unspecified causes; Dermatitis; Atopic dermatitis; Contact dermatitis; Fungal skin diseases; Pruritus; Other skin and subcutaneous diseases; Cataract; Age-related macular degeneration; Refraction disorders; Near vision loss; Other vision loss; Benign prostatic hyperplasia; Mild hearing loss; Moderate hearing loss; Moderately severe hearing loss; Severe hearing loss; Profound hearing loss; Complete hearing loss; Mild heart failure; Moderate heart failure; Severe heart failure; Treated heart failure; Moderate vision loss; Severe vision loss; Blindness; Presbyopia; Road injuries; Pedestrian road injuries; Cyclist road injuries; Motorcyclist road injuries; Motor vehicle road injuries; Other transport injuries; Other exposure to mechanical forces; Non-venomous animal contact.<br>**Lower order causes chosen (n=6)**<br>Enteric infections; Cardiovascular diseases; Diabetes and kidney diseases; Sense organ diseases; Musculoskeletal disorders; Unintentional injuries.
**Implausible cases (n=16)**<br>Cirrhosis and other chronic liver diseases due to hepatitis C; Transport injuries; Fire, heat, and hot substances; Exposure to mechanical forces; Animal contact; Foreign body; Environmental heat and cold exposure; Other unintentional injuries; Physical violence by other means; Lead exposure; Occupational exposure to asbestos; Diet low in legumes; Diet low in whole grains; Diet high in processed meat; Diet high in trans fatty acids; High body-mass index.
**Included items (n=36)**<br>Diarrheal diseases; Protein-energy malnutrition; Neoplasms; Ischemic heart disease; Stroke; Non-rheumatic valvular heart disease; Cardiomyopathy and myocarditis; Atrial fibrillation and flutter; Peripheral artery disease; Other cardiovascular and circulatory diseases; Heart failure; High systolic blood pressure; Chronic respiratory diseases; Peptic ulcer disease; Gallbladder and biliary diseases; Alzheimer's disease and other dementias; Parkinson's disease; Major depressive disorder; Diabetes mellitus; Chronic kidney disease; Skin and subcutaneous diseases; Hearing loss; Blindness and vision impairment; Other sense organ diseases; Rheumatoid arthritis; Osteoarthritis; Low back pain; Gout; Low bone mineral density; Urinary diseases and male infertility; Genital prolapse; Endocrine, metabolic, blood, and immune disorders; High LDL cholesterol; Edentulism and severe tooth loss; Falls; Low physical activity.

Table S3. Lists and categorisation of the items in the GBD-FI and 10 other frailty indexes.

Frailty index (number of items)	Biological	Cognition	Mental wellbeing	Nutrition/weight	Disability/Function	Other
**Evaluative Frailty Index for Physical Activity (EFIP; n=50)**	N=12<br>Are you dizzy when standing up?; Do you have COPD / breathing problems?; Do you have CVA / stroke in the past?; Do you have diabetes / diabetes?; Do you have heart failure?; Do you have high blood pressure?; Do you have pain in the musculoskeletal system (muscles/joints)?; Do you have problems in the musculoskeletal system?; Do you have problems seeing?; Do you have problems with the cessation of urine and / or feces (incontinence)?; Do you have trouble hearing?; Do you take more than four medications daily?	N=3<br>You always know what day and / or time it is?<br>Do you have trouble remembering appointments?<br>Do you have trouble remembering names of family / friends?	N=7<br>Do you feel that everything you takes effort and you have to turn yourself committed to do something?<br>Do you feel sad / depressed?<br>Do you feel generally happy?<br>Do you feel nervous or anxious?<br>Are you afraid of falling?<br>Do you feel lonely?; If you have little energy / you feel tired?	0	N=23<br>Are there tasks that others do for you that you did before yourself?; Are you experiencing barriers in undertaking outdoor activities (eg regarding transport)?; Are you experiencing problems in your living situation?; Are you going to avoid activities in the last month?; Do you need help climbing stairs?; Do you need help getting dressed?; Do you need help getting out of an ordinary chair?; Do you need help going to the toilet?; Do you need help to sit on a regular chair?; Do you need help with moving in bed?; If you move at least 30 minutes each day so that you are getting warm / light sweat?; If you need help doing errands?; If you need help, there are people who can and want to help?; Is there enough professional help / support?; Need help arrive at the check out of bed?; Need help with housework?; Need help with medication?; Need help with showering?; There are plenty of activities in your area where you can take part?; Use aids (cane / walker) when walking?; Will you at least 1 time per week independent outdoors?; You can walk around independently at home?; You can walk around independently outside?	N=5<br>Did you undergo an unplanned hospital admission in the last three months?; Has your health in the past year? If so, how?; Have you fallen in the last 6 months?; How do you rate your own health?; How do you rate yourself in your fitness?
**Frailty Index from Searle et al. (SearleFI, n=40)**	N=10<br>Arthritis; Cancer; CHF; Chronic Lung Disease; Diabetes; Heart attack; High blood pressure; Peak Flow ; Stayed in bed at least half the day due to health (in last month); Stroke	N=1<br>MMSE	N=5<br>Feel Depressed; Feel Everything is an Effort; Feel Happy; Feel Lonely; Have Trouble getting going	N=2<br>BMI ; Lost more than 10 lbs in last year	N=20<br>Cut down on Usual Activity (in last month); Grip Strength ; Help Bathing; Help Dressing; Help Eating; Help getting in/out of Chair; Help Grooming; Help lifting 10 lbs; Help Shopping; Help taking Medication; Help up/down Stairs; Help Using Toilet; Help Walking around house; Help with Finances; Help with Housework; Help with meal Preparations; Rapid Pace; Shoulder Strength; Usual Pace; Walk outside	N=2<br>How health has changed in last year ; Self-rated of Health;
**Frailty Index Beijing Longitudinal Study of Ageing (FIBLSA, n=33)**	N=12<br>Arthritis; Cataract; Coronary heart disease; Do not hear clearly; Glaucoma; Hypertension; Stroke; Thyroid diseases; TIA/small stroke; Tremor; Urinary incontinence; Wear a hearing aid	N=1<br>MMSE score	N=5<br>Do not feel a lot of fun in life; Do not feel very happy; Do not have much energy; Feel less useful; Feel there is nothing to do	0	N=15<br>Need any other personal care; Need help in running housework; Need help with bathing; Need help with cooking meals; Need help with dressing; Need help with eating; Need help with getting on/off bed; Need help with grooming; Need help with managing money; Need help with moving in house; Need help with shopping; Need help with taking a bus; Need help with up/down stairs; Need help with walking 300 m; Use a walking stick	0
**Comprehensive Geriatric Assessment frailty index (FI-CGA, n=11)**	N=4<br>Bladder; Bowel; Communication ; Comorbidity	N=1<br>Cognitive	N=1<br>Mood	N=1<br>Nutrition	N=4<br>Balance; Instrumental activities of daily living/activities of daily living; Mobility ; Social	0
**70-item Frailty Index (FI70, n=70)**	N=26<br>Arthritis; Asthma; Biting on hard foods; Breathlessness; Cancer; Cataracts; Chronic lung disease; Dentures; Diabetes or high blood sugar; Dizziness; Eyesight; Hearing; Heart attack; Heart trouble or angina; High blood cholesterol; High blood pressure; Incontinence; Long-term illness; Osteoporosis; Pain in any joint; Parkinson disease; Persistent cough; Stomach or duodenal	N=5<br>Concentration; Delayed recall test; Mathematical performance; Orientation; Verbal fluency score	N=9<br>Depression; Enjoyment; Fatigue; Fear of falling down; Interest; Pessimism; Sleep; Sleeping problems; Suicidality	N=1<br>Appetite	N=25<br>Bathing or showering; Climbing several flights of stairs; Doing work around the house or garden; Dressing; Eating; Getting in or out of bed; Getting up from a chair after prolonged sitting; Lifting or carrying weights over 10 pounds/5 kilos; Limitations with activities; Making telephone calls; Managing money; Moderate Activities; Picking up a small coin from a table; Preparing a hot meal; Pulling or pushing large objects; Reaching or extending arm above shoulder level; Shopping for groceries; Sitting for about two hours; Stooping, kneeling, or crouching; Taking medications; Using a map to figure out how to get	N=4<br>Falling down; Hip or femoral fracture ; In hospital last 12 months; Self-rated health

	ulcer; Stomach or intestine problems; Stroke or cerebral vascular disease; Swollen legs				around; Using the toilet; Vigorous activities; Walking 100 metres; Walking across a room	
**National Long Term Care Survey Frailty Index (NLTCS, n=32)**	N=15<br>Arthritis; Diabetes; Flu; Glaucoma; History of heart attack; History of stroke; Hypertension; Parkinson's disease; Problems with ear; Problems with feet ; Problems with hearing; Problems with teeth; Problems with vision; Stomach problem; Trouble with bladder/bowels	N=1<br>Dementia	0	0	N=13<br>Difficulty with cooking; Difficulty with dressing; Difficulty with eating; Difficulty with getting bath; Difficulty with getting in/out bed; Difficulty with going out; Difficulty with light house work; Difficulty with managing money; Difficulty with shopping; Difficulty with taking medicine; Difficulty with toileting; Difficulty with using telephone; Difficulty with walk around	N=3<br>Broken bones; Broken hip; Self-rated health
**Electronic frailty index (eFI, n=36)**	N=26<br>Anaemia and haematinic deficiency; Arthritis; Atrial fibrillation; Cerebrovascular disease; Chronic kidney disease; Diabetes; Dizziness; Dyspnoea; Foot problems; Hearing impairment; Heart failure ; Heart valve disease; Hypertension; Hypotension/syncope ; Ischaemic heart disease; Osteoporosis; Parkinsonism and tremor; Peptic ulcer; Peripheral vascular disease; Polypharmacy; Respiratory disease; Skin ulcer; Thyroid disease; Urinary incontinence ; Urinary system disease; Visual impairment	N=1<br>Memory and cognitive problems	N=1<br>Sleep disturbance	N=1<br>Weight loss and anorexia	N=5<br>Activity limitation; Housebound ; Mobility and transfer problems; Requirement for care ; Social vulnerability	N=2<br>Falls ; Fragility fracture
**Global Burden of Disease frailty index (GBD-FI, n=36)**	N=31<br>Atrial fibrillation and flutter; Blindness and vision impairment; Cardiomyopathy and myocarditis; Chronic kidney disease; Chronic respiratory diseases ; Diabetes mellitus; Diarrheal diseases; Edentulism and severe tooth loss; Endocrine, metabolic, blood, and immune disorders; Gallbladder and biliary diseases; Genital prolapse; Gout; Hearing loss; Heart failure ; High LDL cholesterol; High systolic blood pressure; Ischemic heart disease; Low back pain; Low bone mineral density; Neoplasms; Non-rheumatic valvular heart disease; Osteoarthritis; Other cardiovascular and circulatory diseases; Other sense organ diseases ; Parkinson's disease; Peptic ulcer disease; Peripheral artery disease; Rheumatoid arthritis; Skin and subcutaneous diseases; Stroke; Urinary diseases and male infertility	N=1<br>Alzheimer's disease and other dementias	N=1<br>Major depressive disorder	N=1<br>Protein-energy malnutrition	N=1<br>Low physical activity	N=1<br>Injuries from falls
**Multimorbidity frailty index (mFI, n=32)**	N=31<br>Asthma; Cardiac dysrhythmias; Chronic airways obstruction, not elsewhere classified; Chronic bronchitis; Chronic renal failure; Contact dermatitis and other eczema; Diseases of esophagus; Disorders of function of stomach; Duodenal ulcer; Functional digestive disorders, not elsewhere classified; Gastric ulcer; General symptoms; Heart failure; Hyperplasia of prostate; Hypertensive heart disease; Late effects of cerebrovascular disease; Occlusion of cerebral arteries; Other and ill-defined cerebrovascular	N=1<br>Senile and presenile organic psychotic conditions	0	0	0	0

	disease; Other and unspecified anemias; Other cellulitis and abscess; Other disorders of bone and cartilage; Other disorders of eyelids; Other disorders of urethra and urinary tract; Other forms of chronic ischemic heart disease; Other noninfectious gastroenteritis and colitis; Parkinson's disease; Pneumonia, organism unspecified; Pruritus and related conditions; Symptoms involving cardiovascular system; Symptoms involving urinary system; Vertiginous syndromes and other disorders of vestibular system					
**Modified frailty index (mFI, n=11)**	N=10<br>Cerebrovascular accident or stroke with neurologic deficit; Congestive heart failure within 30 days before surgery; Diabetes mellitus (noninsulin or insulin); History of COPD or current pneumonia ; History of myocardial infarction within past 6 months before surgery; History of transient ischemic attack or cerebrovascular accident with no residual deficits; Hypertension requiring medication; Impaired sensorium; Percutaneous coronary intervention or cardiac surgery or angina 1 month before surgery; Revascularization or amputation due to peripheral vascular disease or rest pain or gangrene	0	0	0	N=1<br>Functional health status before surgery (either partially or totally dependent)	0
**Laboratory frailty index (FI-LAB, n=23)**	N=23<br>Albumin (g/L); AST (SGOT; IU/L); BP, supine systolic (mmHg); BP, supine diastolic (mmHg); Calcium (mM); Creatinine (µM); Folate (nM); Folate, RBC (nM); Glucose, fasting (mM); Hemoglobin (g/L) b; Mean corpuscular volume (fL); Phosphatase, alkaline (IU/L); Phosphorus, inorganic (mM); Potassium (mM); Protein, total (g/L); Sodium (mM); TSH (µIU/L); Thyroxine (T4; nM); T4, Free (pM); Urea (mM); VDRL; Vitamin B12 (pg/L); White blood cells (number/L)	0	0	0	0	0

Figure S1. Visual presentation of the items in (a) the Global Burden of Disease frailty index (GBD-FI) and (b) the electronic frailty index (eFI).

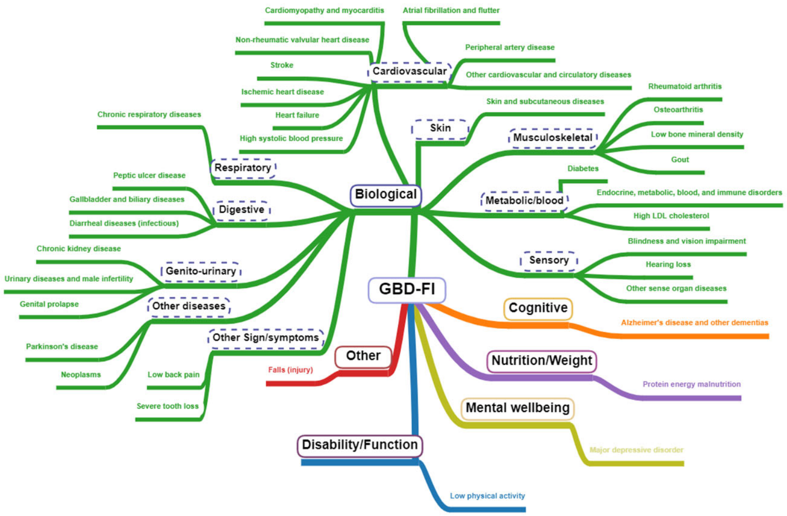

(a)

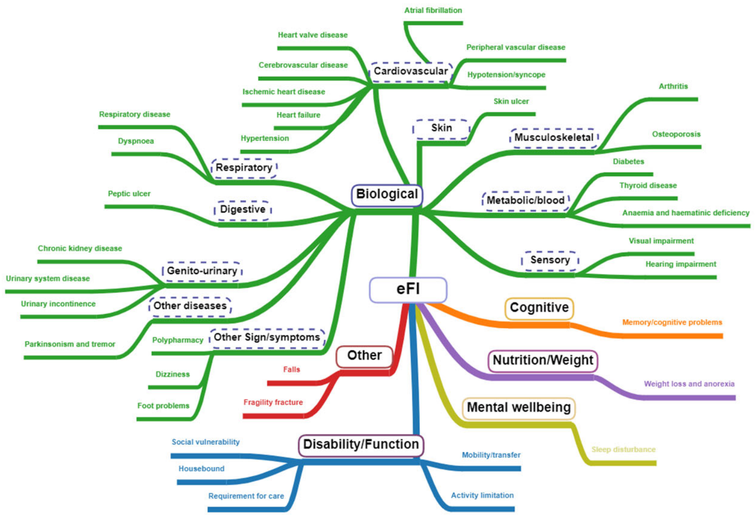

(b)
